# Novel analytical perspectives on nonlinear instabilities of viscoelastic Bingham fluids in MHD flow fields

**DOI:** 10.1038/s41598-024-78848-8

**Published:** 2024-11-21

**Authors:** Galal M. Moatimid, Yasmeen M. Mohamed

**Affiliations:** Department of Mathematics, Faculty of Education, Ain Shams University, Roxy, Cairo, Egypt

**Keywords:** Nonlinear instability, Bingham fluid, Porous media, Non-perturbative approach, He’s frequency formula, Engineering, Mathematics and computing, Physics

## Abstract

The nonlinear stability of a plane interface separating two Bingham fluids and fully saturated in porous media is inspected in the existing work. The two fluids are compressed by a normal magnetic field. The two fluids have diverse viscoelasticity, densities, magnetic, and porosity medium, with the existence of surface tension at the interface. The motivation of applied physics and engineering relations has encouraged the discussion of the current paper. Because the mathematical behavior is rather complex, the viscoelasticity involvement is reproduced only at the surface of separation, which is well-known as the viscous potential theory. Thereby, the equations of movement are scrutinized in a linear form, whereas a set of nonlinear boundary conditions are supposed. This procedure produces a nonlinear expressive nonlinear partial differential equation of the interface displacement. The non-perturbative approach which is based on the He’s frequency formula is employed to transform the nonlinear distinguishing ordinary differential equation with complex coefficients into a linear one. A novel process relying on the non-perturbative approach is utilized to examine the nonlinear stability and scrutinize the interface presentation. A non-dimensional analysis produces several dimensionless physical numerals. To validate the new approach, a comparison between the non-perturbative approach and its corresponding linear ordinary differential equation via the Mathematica Software is described and interpreted through a set of diagrams. Additionally, the Polar graphs have been elucidated. It is found that the mechanism of the stability does not change in the cases of real and complex coefficients.

**Supplementary Information:**

The online version contains supplementary material available at 10.1038/s41598-024-78848-8.

## Introduction

The Bingham fluid (BF) is a prominent and actively researched subject in particle techniques. These fluids are found in a diverse range of usage, including environments such as blood, mud, and ice, as well as manufacturing settings such as lubricating grease, fresh concrete, polymers, and paints. Bingham plastic fluid and the Bingham pseudo plastic fluid are a type of non-Newtonian liquid that exhibits unique yield stress. A physically consistent particle technique was used to develop and evaluate the BF simulation model^1^. A mathematical investigation was conducted on the flow of a BF driven by a multi-membrane pumping mechanism across porous media^2^. The study explored the effects of incorporating ion-slip characteristics in magnetohydrodynamics (MHD) phenomena on the drift of BF when inflowing between two porous plates in the suction condition^3^. The study investigated the stability of laminar Bingham-Poiseuille inflows in a fluid sheet inflowing down an incline with a constant slope angle^4^. The study examined the flow behavior of the BF in oblique channel conditions and investigated the stabilizing impact of the Bingham parameter. A mathematical solution was obtained for the unsteady inflow of a BF in a channel^5^. The movement of yield stress fluids in porous media exhibits intriguing complexities due to the interaction involving nonlinear rheology and medium heterogeneity^6^. The statistics of non-flowing surfaces were characterized by avalanches initiated at one end. The BF displayed both yield stress and plastic viscosity. The study proposed a numerical solution for the velocity and temperature fields in the time-dependent Couette-Poiseuille inflow of BF in channels^7,8^.

The Hall current was facilitated by electron transport between Landau levels, instigated by the electric field (EF) and magnetic field (MF) components of an electromagnetic wave^9^. The study examined the movement of viscoelastic liquid through an irregular microchannel while considering changes in viscosity and the presence of a porous media^10^. The study focused on analyzing the inflow and energy transport properties of viscoelastic liquids^11^. The study examined the behavior of a layer of Oldroydian viscoelastic liquid. The onset and stability of a triple cross-diffusive viscoelastic fluid layer was investigated^12^. The rheology of viscoelastic fluid was approximated by the nonlinear Oldroyd-B constitutive equation which encompasses Maxwell and Newtonian fluid models as special cases. The stability of a triply diffusive viscoelastic fluid layer in which the fluid density depends on three stratifying agencies possessing different diffusivities was investigated^13^. The viscoelastic fluid is modeled by means of the Oldroyd-B constitutive equation. Analytical expressions were obtained for steady and oscillatory onset by carrying out the linear instability analysis and the crossover boundary between them was demarcated by identifying a dimension-two point in the viscoelastic parameters plane. The triple diffusive convection in an Oldroyd-B fluid-saturated porous layer was investigated by performing linear and weakly nonlinear stability analyses^14^. The condition for the onset of stationary and oscillatory was derived analytically. Contrary to the observed phenomenon in Newtonian fluids, the presence of viscoelasticity of the fluid degenerates the quasi-periodic bifurcation from the steady quiescent state. To reach to explicit characteristic equation, the present work follows a simplified approach. The study examined the impact of the Soret numeral on the mixed convection inflow over a vertical surface, using MHD as a factor^15^. A unique model examining the influence of Hall current on an elastic semiconductor medium was investigated^16^. The intersection of the MF and the micro elements (microstructure) of the medium was examined. The study investigated the transient behavior of hydromagnetic nanofluid flow between two vertically aligned magnetized surfaces that enclose a porous medium^17^. The flow was influenced by both the induced MF and the heat conduction effects.

The use of the nonlinear instability concept proved crucial in comprehending and predicting intricate atmospheric phenomena. These may be hurricanes, tornadoes, and other turbulent weather events. The significance of nonlinear vibrations in disciplines such as physics, electrical engineering, and modern manufacturing is well acknowledged. They serve as the essential basis for comprehending diverse nonlinear phenomena. The Duffing oscillator (DO) played a pivotal role in this particular circumstance. Their responses illustrated a diverse array of natural processes, physical notions, and engineering occurrences. Several suggestions are given on the conceptual aspects of the DO problem. The solution to the DO, which experiences a decrease in stiffness, for a system without external forces and with damping, was expressed in terms of the Jacobi elliptic functions^18^. Therefore, several researchers attempted to determine the solution. The damped ordinary differential equation ODE with larger-order nonlinearities was solved by applying a combination of arithmetic and conceptual procedures^19^. The comparison between He’s frequency formula (HFF) and its modifications was undertaken concerning residual computation^20^. The understanding of HFF was vital for the analysis of the other elements in the prediction results^21^. Although an accurate response could be obtained, there was still room for additional advancement. The inclusion of considerable nonlinearity in ODEs was designed to showcase the precision of the solution approach^22^. The un-damped degree of oscillation was significantly enhanced by the high-frequency force field^23^. The non-perturbative approach (NPA) has recently been employed in the dynamic system. Both the domains of dynamical systems and Electrohydrodynamic stability have employed the NPA to conduct stability analyses^24–35^. Given the challenges in evaluating the DO, this topic has been acknowledged as a critical field of research that requires further investigation to generate more accurate responses. Thus, in the current study, the use of the NPA is considered to be satisfactory. The HFF was employed to analyze nonlinear vibrations in mechanical systems, a crucial determinant in the development of robust and dependable mechanical structures and components.

The non-Newtonian liquids are essential for chemicals, technological advancement, and manufacturing. Therefore, discussing these fluids is becoming more and more predominant. Consequently, the goal of this study is to evaluate the nonlinear stability of the BF. The viscous potential theory (VPT) is generally used in the mathematics treatment to make simpler the calculation. The comprehension of interface stability in viscoelastic non-Newtonian fluids is crucial in the fields of metallurgical and polymer manufacturing. The hydrodynamic characteristics of non-Newtonian fluid flow, such as blood analogs in artificial structures, can significantly impact the stability and function of biomedical equipment. In conclusion, the Lorentz force on the interface stability between two viscoelastic non-Newtonian BF is an intricate phenomenon that can greatly impact the dynamics of the system. By employing linear stability analysis and comprehending the altered electromagnetic forces, we may anticipate and regulate stability in a wide range of applications, encompassing industrial processes and natural geophysical flows. A uniform normal MF is applied to the upper and lower fluids. A conception for the perturbed interface characteristic is also developed. Straightforward viscoelastic fluid flows with constant comparable speeds are the essential topic of the recent analysis. Nevertheless, a weak nonlinear strategy, which relies on the movement of calculating the linear formulas of movement under the pertinent boundary conditions (BCs), has been deemed. During this strategy’s fundamental, the NPA has been the essential topic of this investigation. The remarkable, expressive, and intriguing process for treating numerous categories with nonlinear dynamics is seen by this inventive procedure. As was formerly recorded in the context of nonlinear stability analysis, conventional approaches frequently have outcomes in Schrödinger & Landau Ginzburg formulas. It ought to be noted that the preceding nonlinear stability was tackled in an intricate procedure. On the antithesis, the recent NPA verifies and promotes simple stability standards. The article is organized as. In § 2, we present a mathematical formation of our MHD issue. The necessary BCs and dominant formula of movement are covered in this section. Furthermore, the nonlinear characteristic formula is achieved in § 3. The case of the complex factors of the nonlinear displacement equation is involved in § 4. There are diverse circumstances in this area. Our essential key outcomes are summarised in § 5.

## Construction of the problem

**Fig. 1:**
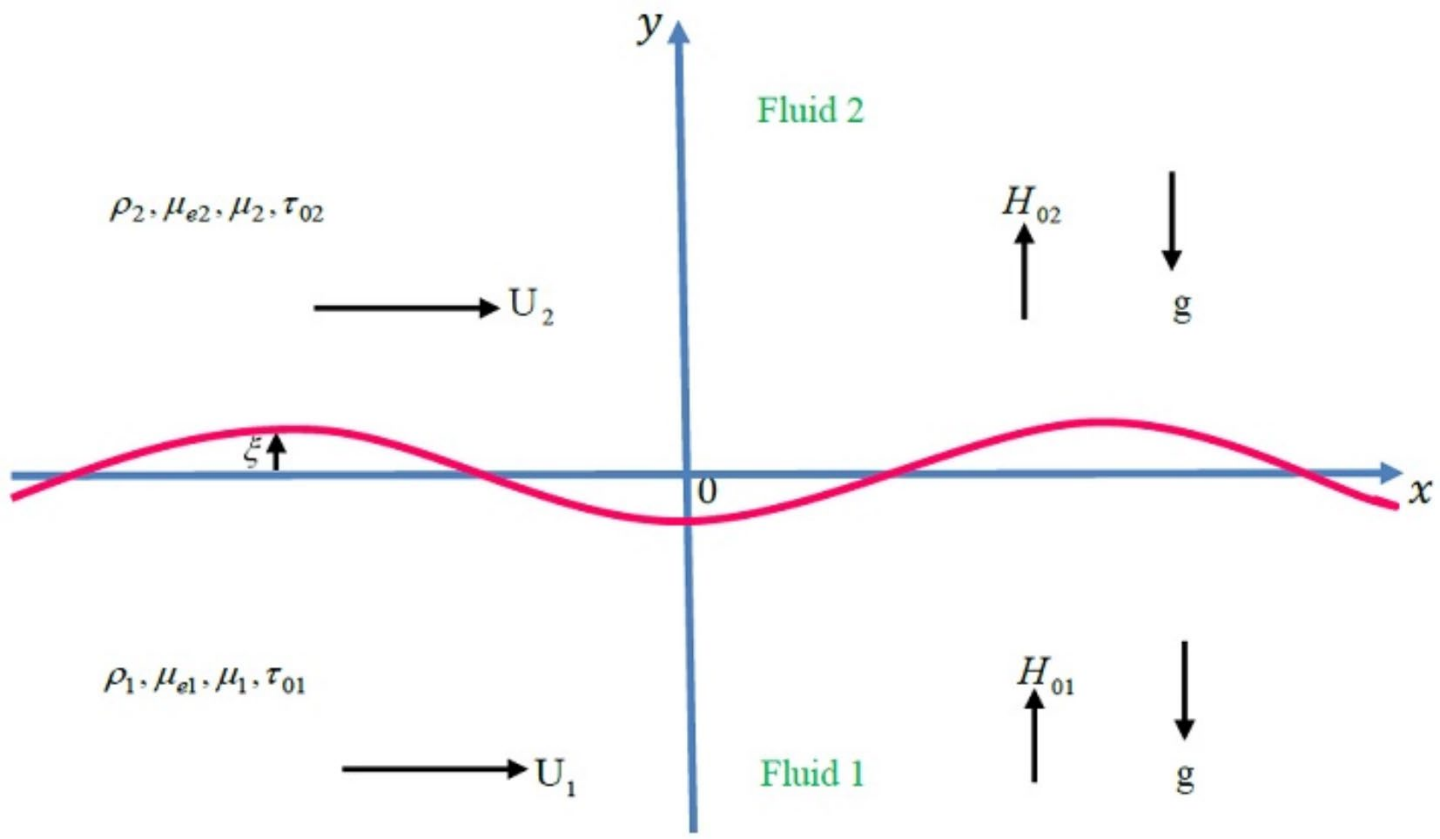
Clarifies the physical model of the structure.

Fig. 1 demonstrates the flow dynamics of two semi-infinite horizontal fluid layers within a permeable medium. The fluids are regarded in regions 1 and 2 across the areas $y < 0$ and $y> 0$ are viscoelastic, incompressible liquids. The inflow of liquids in porous media is governed by Darcy’s law. An empirical principle that links the permeability of the medium and the liquid’s viscosity to its flow rate through that medium is supposed. This law plays a key role in scenarios where slow, creeping flows dominate. Capillary action or biological systems, where the movement of fluids, like blood or nutrients, is controlled by viscosity and the medium’s permeability are a few examples. In this context, permeability is presupposed to be constant for both non-Newtonian liquids, simplifying the mathematical analysis. Physically, Darcy’s law serves as a crucial bridge between liquid dynamics and physiological processes. The system is influenced by gravitational acceleration, denoted as $\underline{g} = - g\underline{e}_{y}$, where $\underline{e}_{y}$ is the unit vector along the vertical $y -$ axis. The analysis is conducted using a two-dimensional Cartesian coordinate system $(x,\,y)$, with the $x -$ axis aligned horizontally between the two fluid layers and the $y -$ axis oriented vertically. The fluids in the lower and upper layers have densities $\rho_{1}$, $\rho_{2}$ and magnetic permeabilities $\mu e_{1}$ and $\mu e_{2}$. The viscoelastic properties of these fluids are modeled by employing the BF framework. The subscripts 1 and 2 are utilized to distinguish between the lower and upper liquids, respectively. The two liquid layers are stream with velocities $U_{1}$ and $U_{2}$ in the positive $x -$ direction. A uniform MF is applied perpendicular to the inflow, represented by $\underline{B}_{j} = \left( {0,\,\,B_{0j} } \right)=\mu_{ej}\left( {0,\,\,H_{0j} } \right)$. The induced MF is neglected based on the assumption that the magnetic Reynolds numeral is small; meaning the impact of the fluid’s movement on the MF is minimal. Consequently, the MFs within the lower and upper liquids are $H_{01}$ and $H_{02}$, respectively.

Based on the principle that the forces acting on a volume element of liquid must be in equilibrium, the local volume-averaged balance of linear momentum is derived. This formulation, which ensures that the net force on the liquid element is zero, follows the approach outlined in former studies^32,36–38^

1 $$\rho_{j} \left[ {\frac{{\partial \underline{V}_{j} }}{\partial t} + \left( {\underline{V}_{j} \cdot \nabla } \right)\underline{V}_{j} } \right] = - \nabla P_{j} + \nabla \cdot \underline{S} - \rho_{j} \underline{g} \underline{e}_{y} + \underline{J} \wedge \underline{B} - \frac{{\mu_{j} }}{\beta }\underline{V}_{j} ,\,j = 1,2,$$

and

2 $$\nabla \cdot \underline{V}_{j} = 0.$$

The fundamental theory of motion, as described in Eq. (1), is derived by utilizing the VPT, which is based on several key factors:

The Euler equation is derived from the Navier-Stokes equations using the framework of the VPT, as detailed in^28–35^. In deriving the inflow equation, the stress tensor for the viscous fluid is omitted from the starting equation Eq. (1). The main governing equation is formulated as a mathematical model incorporating the Brinkman-Darcy law, which accounts for fluid flow in porous media. Within the context of VPT, fluids are considered irrotational, meaning that their flow lacks rotational motion. A similar approach has been successfully used to transition from perturbation methods to analyzing viscoelastic fluids. In this study, the derivations align perfectly with the principles of VPT. Since the equations governing irrotational flow can be derived from Laplace’s equation, it becomes possible to adjust the BCs to include basic viscoelastic effects. The viscoelastic impacts in this work are specifically addressed by applying BCs related to normal stress. The key governing equations for common fluid phases are derived under the assumption of viscoelastic behavior, providing a comprehensive understanding of the fluid dynamics involved.

The equations of generalized Ohm’s law are expressed as^36–40^:

3 $$\underline{J} = \sigma_{H} \left( {\underline{E} + \underline{V} \wedge \underline{B} } \right),$$

and

4 $$\nabla \cdot \underline{B} = 0,\;{\text{and}}\;\nabla \wedge \underline{E} = \underline{0} .$$

By neglecting the external EF and disregarding the impacts of ionized gas polarization, we presuppose that the EF vector is zero $\left( {\underline{E} = \underline{0} } \right)$. This assumption simplifies the analysis by eliminating external forces and ionization-related complexities. By setting the EF vector to zero, the governing equations describe the movement and conduct of charged particles based purely on internal dynamics. This approach is commonly employed in theoretical studies to streamline complex systems and highlight specific phenomena without external interference.

The interface may be formulated as^28,31,32,34^:

5 $$S_{e} (x,y;t) = y - \xi (x;t).$$

The perpendicular vector $\left. {\underline{n} } \right|_{y = \xi }$ is expressed as^28,31,32,34^:

6 $$\underline{n} = \frac{{\nabla S_{e} }}{{\left| {\nabla S_{e} } \right|}} = \left( { - \xi_{x} \underline{e}_{x} + \underline{e}_{y} } \right)\left( {1 + \xi_{x}^{2} } \right)^{ - 1/2} ,$$

The zero-order pressure is expressed as:

7 $$P_{0j} = - \rho_{j} gy - \left( {\frac{{\mu_{j} }}{\beta } + \sigma_{H} B_{0j}^{2} } \right)\,U_{j} x + C_{0j} ,$$

and

8 $$C_{01} - C_{02} = \tau_{0j} - gy\rho_{j} - \frac{1}{2}\left( {\mu_{e1} H_{01}^{2} - \mu_{e2} H_{02}^{2} } \right) - \left( {\frac{{\mu_{j} }}{\beta } + \sigma_{H} B_{0j}^{2} } \right)U_{j} x.$$

It should be noted that Eq. (8) is accomplished later from the relevant BC.

The entire velocity is introduced in terms of the potential $\Phi_{j}$ as reported in^28,31,32,34^:

9 $$\underline{V}_{j} = {\text{U}}_{j} \underline{e}_{x} + \nabla \Phi_{j} = \frac{{\partial \Phi_{j} }}{\partial y}\underline{e}_{y} + \left( {{\text{U}}_{j} + ik\,\Phi_{j} } \right)\underline{e}_{x} ,$$

and

10 $$\nabla^{2} \Phi_{j} = 0.$$

Following^28,31,34^, any function may be introduced by:

11 $$F^{*} (x,y;t) = \hat{f}(y,t)e^{ikx} + c.c,$$

Thereby, from Eq. (10) one gains:

12 $$\Phi_{1} (x,y;t) = a_{1}^{*} (t)e^{{k\left( {ix + y} \right)}} ,\; - \infty \le y \le \xi ,$$

and

13 $$\Phi_{2} (x,y;t) = a_{2}^{*} (t)e^{{k\left( {ix - y} \right)}} ,\;\xi \le y \le \infty ,$$

where $a_{1}^{*} (t)$ and $a_{2}^{*} (t)$ are determined from the applicable BC.

From Eq. (1) $P_{j}$ may be expressed as:

14 $$P_{j} = - \rho_{j} \Phi_{jt} - \rho_{j} g\,\,y - \left( {\frac{{\mu_{j} }}{\beta } + \sigma_{H} B_{0j}^{2} } \right)\Phi_{j} .$$

In the topic of MHD, it is well understood that a quasi-static approximation can be applied. This approximation implies that the influences of dynamic magnetic forces are deemed minimal, resulting in the MF being presumed irrotational, meaning it has no curl. Consequently, this allows the MF to be expressed in terms of a magnetic scalar potential $\Psi (x,y;t)\,$ that exhibits a gradual variation. According to the governing bulk equations $\left( {\nabla \wedge \underline{H}_{0j} = \underline{0} } \right)$, the magnetic scalar potentials must satisfy Laplace’s formula, which governs their spatial distribution within the system. Thereby, after the disturbance to the surface of separation, the MF is formulated as^31^:

15 $$\underline{B}_{j} = B_{0j} \underline{e}_{y} - \nabla \Psi_{j} ,$$

and

16 $$\nabla^{2} \Psi_{j} = 0.$$

Subsequently $\Psi_{1} (x,y;t)$ and $\Psi_{2} (x,y;t)$ may be exhibited as:

17 $$\Psi_{1} (x,y;t) = b_{1}^{*} (t)e^{{k\left( {ix + y} \right)}} ,\; - \infty \le y \le \xi ,$$

and

18 $$\Psi_{2} (x,y;t) = b_{2}^{*} (t)e^{{k\left( {ix - y} \right)}} ,\;\xi \le y \le \infty ,$$

where $b_{1}^{*} (t)$ and $b_{2}^{*} (t)$ are two-time dependent functions to be ascertained from the relevant BC.

The entire total, hydro, and magnetic stress tensors may be expressed as^31,39,40^:

19 $$\sigma_{ij} = \sigma_{ij}^{hydro} + \sigma_{ij}^{mag} ,$$

20 $$\sigma_{ij}^{hydro} = - P_{j} \delta_{ij} + \left( { - \tau_{0j} + \mu_{j} \mathop \gamma \limits^{ \cdot } } \right)$$

and

21 $$\sigma_{ij}^{mag} = H_{0i} B_{0j} - \frac{1}{2}\mu_{ej} H_{0}^{2} \delta_{ij} .$$

It should be noted that:

- The BF

The BF model characterizes a type of non-Newtonian liquid that acts as a solid under low stress but transitions to a viscous inflow when the applied stress exceeds a specific threshold, referred to as yield stress. This model is commonly applied to materials such as toothpaste, mud, slurries, and various industrial liquids.

The components of the stress tensor $\underline{S}$ for BF may be expressed as^39,40^:

22 $$S_{xx} = - \tau_{0j} + 2\mu_{j} \Phi_{jxx} ,\;S_{xy} = - \tau_{0j} + 2\mu_{j} \Phi_{jxy} = S_{yx} ,\;{\text{and}}\;S_{yy} = - \tau_{0j} + 2\mu_{j} \Phi_{jyy} ,$$

It should be noted that this model involves the conventional Newtonian liquid as a special case when $\tau_{0j} = 0.$

- Difference from other fluids

Newtonian fluids, such as water and air, exhibit a constant viscosity regardless of the applied stress or shear rate, resulting in a linear relationship between stress and strain rate that passes through the origin. In contrast, Bingham fluids have a yield stress, which requires a minimum level of stress to initiate flow. Other types of non-Newtonian fluids include pseudoplastic (shear-thinning) fluids, where the viscosity decreases with increasing shear rate (e.g., blood, polymer solutions), and dilatant (shear-thickening) fluids, where viscosity increases as shear rates rise (e.g. corn starch in water). The BF model is unique in that it incorporates the concept of yield stress, whereas other non-Newtonian liquids generally exhibit a continuous relationship between stress and strain rate without a defined yield point.

- Modifications

Modifications and extensions to the BF model include the Herschel-Bulkley model, which enhances the original model by integrating a power-law component that accounts for more complex inflow performances, thus incorporating both yield stress and non-linear viscosity. This model effectively combines characteristics of Bingham plastics and power-law liquids. Additionally, the Bingham-Papanastasiou regularization is employed in numerical models to manage the sudden transition between rigid and fluid states. These regularized approaches smooth the behavior around the yield stress, resulting in greater stability in computational analyses.

- Real applications

The BF model is widely applicable across diverse industries and areas. In the oil and gas industry, drilling muds function as BFs, providing stability while effectively transporting drill cuttings until a specific stress threshold is surpassed. Similarly, in construction and civil engineering, cement slurries exhibit BF conduct, maintaining their shape until adequate force is employed to initiate inflow. Biological systems, such as blood inflow in microcirculation, can also display BF characteristics due to the arrangement of red blood cells under low shear stress. In consumer products, items like toothpaste and cosmetics demonstrate BF properties, retaining their shape until sufficient pressure is applied to dispense them. Over and above, geophysical phenomena like landslides and mudflows can be analyzed by applying the BF model, as certain soils and muds only begin to flow when the stress exceeds a critical threshold, which aids in predicting and managing these natural occurrences.

### Nonlinear BCs

Accurately determining the hydrodynamic and magnetic stresses is essential for understanding liquid inflow and MF interactions. The BCs required for this calculation are derived from established formulations^28,31,34^, and are critical for properly defining the performance of the system at its boundaries. These BCs provide the necessary restrictions to solve the stress distribution within the system, ensuring the correct application of physical laws governing the interactions between the liquid and the MFs. Thus,

23 $$\xi_{t} + \left( {U_{j} + ik\Phi_{j} } \right)\xi_{x} = \Phi_{jy} ,\;{\text{at}}\;y = \xi$$

24 $$\underline{n} \wedge \underline{H}_{01} = \underline{n} \wedge \underline{H}_{02} ,\;{\text{at}}\;y = \xi .$$

and

25 $$\underline{n} \,.\,\left( {\mu_{e1} \underline{H}_{01} - \mu_{e2} \underline{H}_{02} } \right) = 0, \;{\text{at}}\;y = \xi .$$

### The nonlinear discriminant equation

The formerly mentioned BCs highlight that the perpendicular component of the stress tensor undergoes a discontinuity at the interface due to the presence of surface tension (ST), denoted by a specific symbol $T.$ This discontinuity arises because ST acts along the interface, causing a difference in the stresses across it. Mathematically, this condition can be expressed as detailed in references^30–35^. The ST, as the force acting at the boundary between two liquids or phases, introduces this jump in the stress tensor. The BCs account for this by properly describing the balance between the fluid forces and ST at the interface. Thereby,

26 $$\underline{n} \,.\,\left( {\underline{F}_{1} - \underline{F}_{2} } \right) = T\nabla \cdot \underline{n} ,\;{\text{at}}\;y = \xi ,$$

and

27 $$\underline{F} = \left( {\begin{array}{*{20}c} {\sigma_{xx} } & {\sigma_{xy} } \\ {\sigma_{yx} } & {\sigma_{yy} } \\ \end{array} } \right)\left( {\begin{array}{*{20}c} {n_{x} } \\ {n_{y} } \\ \end{array} } \right),$$

The particular findings of Eqs. (12), (13), (17), and (18) with (23-25) are presupposed as:

28 $$\Phi_{1} = \frac{{\left( {\xi_{t} + U_{1} \xi_{z} } \right)}}{{k\left( {1 - i\xi_{x} } \right)}}e^{{k\left( {y - \xi } \right)}} ,$$

29 $$\Phi_{2} = - \frac{{\left( {\xi_{t} + U_{2} \xi_{z} } \right)}}{{k\left( {1 + i\xi_{x} } \right)}}e^{{k\left( {\xi - y} \right)}} ,$$

30 $$\Psi_{1} = - \frac{{i\,\mu_{e2} \,\left( {H_{01} - H_{02} } \right)\,\xi_{x} \,\,e^{{k\left( {y - \xi } \right)}} }}{{k\left( {1 - i\xi_{x} } \right)\left( {\mu_{e1} + \mu_{e2} } \right)}},$$

and

31 $$\Psi_{2} = \frac{{i\,\mu_{e1} \,\left( {H_{01} - H_{02} } \right)\,\,\xi_{x} \,\,e^{{k\left( {\xi - y} \right)}} }}{{k\left( {1 + i\xi_{x} } \right)\left( {\mu_{e1} + \mu_{e2} } \right)}}.$$

Thereby, $P_{1} ,\,\,\,P_{2}$ may be attained as:

32 $$P_{1} (x,y;t) = - \rho_{1} gy - \frac{{e^{{k\left( {y - \xi } \right)}} }}{{k\left( {1 - i\xi_{x} } \right)}}\left( {\rho_{1} \left( {\xi_{tt} + U_{1} \xi_{xt} } \right) + \left( {\frac{{\mu_{1} }}{\beta } + \sigma_{H} B_{0j}^{2} } \right)\left( {\xi_{t} + U_{1} \xi_{x} } \right)\,} \right)\,\,\,\,\,\,\,\, - \infty \le y \le \xi ,$$

and

33 $$P_{2} (x,y;t) = - \rho_{2} gy + \frac{{e^{{k\left( {\xi - y} \right)}} }}{{k\left( {1 + i\xi_{x} } \right)}}\left( {\rho_{2} \left( {\xi_{tt} + U_{2} \xi_{xt} } \right) + \left( {\frac{{\mu_{2} }}{\beta } + \sigma_{H} B_{0j}^{2} } \right)\left( {\xi_{t} + U_{2} \xi_{x} } \right)} \right),\,\,\,\,\,\,\xi \le y \le \infty .$$

To achieve this, a non-dimensional approach is employed. This involves introducing non-dimensional parameters to simplify the problem and make it more manageable. The specific non-dimensional factors used in this context are defined as follows:

$$W_{e} = \rho_{1} U_{1}^{2} L/T$$, $$D_{a} = \beta /L^{2}$$, $$B_{d} = \rho_{1} \,g\,\,L^{2} /T$$, $$Ha^{2} = \sigma_{H} L^{2} B_{0j}^{2} /\mu_{1} ,$$$$\xi^{*} = \xi /L$$, $$B_{g} = \tau_{01} \,L\left( {\mu_{1} U_{1} } \right)^{ - 1} ,$$ $$Z = \mu_{1} /\sqrt {\rho_{1} \,T\,L}$$, $$H_{0}^{*2} = L\,\mu_{e1} \,H_{0}^{2} /T$$, $$t^{*} = \sqrt {T/\rho_{1} \,L^{3} } t$$, $$\rho^{*} = \rho_{2} /\rho_{1}$$, $$\mu_{e}^{*} = \mu_{e2} /\mu_{e1}$$, $$U^{*} = U_{2} /U_{1}$$, $$\mu^{*} = \mu_{2} /\mu_{1}$$, and $$\tau_{y}^{*} = \tau_{y2} /\tau_{y1}$$. The star notation has been omitted for simplicity.

Since this study focuses on temporal instability, we will differentiate with respect to the coordinate to transform the partial differential equation (PDE) into an ODE. By taking derivatives with respect to $x$ and setting the result equal to zero, we simplify the problem. Essentially, we can consider an observer located at a fixed position $x = 0$, who tracks the evolution of the interface displacement wave over time. This approach allows us to derive an ODE that characterizes the behavior of the surface displacement.

Consequently, the non-dimensional ODE may be expressed as:

34 $$\xi^{\prime \prime } + (r_{1} + ir_{2} )\,\xi^{\prime } + (r_{3} + ir_{4} )\,\xi + r_{5} \,\xi \xi^{\prime \prime } + (r_{6} + ir_{7} )\xi \xi^{\prime \prime } + (r_{8} + ir_{9} )\,\,\xi^{2} \, + r_{10} \,\xi^{2} \xi^{\prime \prime } + (r_{11} + ir_{12} )\xi ^{\prime}\xi^{2} + (r_{13} + ir_{14} )\xi^{3} = 0,$$

The factors $r_{1} \to r_{14}$ are involved in the Online Appendix.

## Methodology

The stability analysis is conducted by using NPA under two different scenarios: initially, when $W_{e} = 0$ is encountered, and subsequently when $W_{e} \ne 0.$ is met. This method allows us to evaluate the stability of the system under these distinct restrictions, providing insights into how each scenario affects the overall stability. In the situation where the Weber numeral equals zero $\left( {W_{e} = 0} \right)$, it signifies that ST forces are vastly more significant than inertial forces in the fluid. In this context, the impact of ST is so pronounced that it dictates the shape and stability of droplets or bubbles with exceptional precision. This means that even in the presence of disturbances or external forces, the ST is sufficient to preserve the original shape of the droplets or bubbles with minimal distortion. The ST thus becomes the predominant factor influencing the behavior of the fluid, overshadowing any effects due to the fluid’s bulk motion or inertia. Consequently, in this regime, the dynamics of the fluid are governed almost entirely by the effects of surface tension, with inertial forces playing an insignificant conduct. Overall, the Weber numeral $W_{e}$ is crucial for understanding and predicting the dynamics and stability of liquid systems by evaluating the relative balance between inertial forces and ST forces. It has three essential applications in liquid dynamics:

i. High $$\left( {W_{e}>> 1} \right)$$ i.e. $$\left( {W_{e} \ne 0.} \right)$$

This suggests that, in this situation, inertial forces are more dominant than ST forces. As a result, this understanding is crucial for determining droplet sizes in sprays and for optimizing the breakup of fuel droplets. Improved droplet breakup enhances fuel atomization, which is the key to optimizing engine performance. Better atomization results in more complete combustion and efficient fuel usage, leading to improved engine efficiency and reduced emissions.

ii. Low $$\left( {W_{e} < < 1} \right)$$ i.e. $$\left( {W_{e} \to 0} \right)$$

This indicates that in this scenario, ST plays a more significant conduct than inertial forces. Consequently, this insight is critical for designing devices where exact control over droplet formation is necessary. For instance, in inkjet printing technology, ST dictates the behavior of ink droplets, including their formation, expulsion, and trajectory. By applying this understanding, engineers and designers can refine the printing process to ensure that ink droplets are generated with consistent size, shape, and velocity. Such precision is essential for achieving high-quality prints, as it guarantees that each droplet is deposited with accuracy and uniformity on the paper or other media. This results in clearer, sharper images and text, and overall improved print quality, as well as enhanced reliability and performance of the printing device.

iii. Weber numeral near unity $\left( {W_{e} \approx 1} \right)$

This signifies that inertial forces and ST forces are in a state of equilibrium, meaning their impacts on droplet behavior are balanced. This balance significantly influences the dynamics of droplets, leading to complex and diverse behavior patterns. Understanding this equilibrium is essential in analyzing how sprays change between the different operational regimes, such as transitioning from producing a fine mist to forming larger droplets. This knowledge is particularly important in practical applications like fuel injection systems, where controlling droplet size impacts combustion efficiency. Additionally, this insight is valuable for studying the behavior of systems with particles and bubbles, such as in chemical reactors or wastewater treatment. It helps in understanding phenomena like particle dispersion, bubble formation, and stability, ultimately contributing to more efficient and effective design and operation of these systems in various industrial and environmental contexts.

Over and above, the Weber numeral is pivotal in assessing the stability and interactions of bubbles and foams within multiphase liquid systems. It quantifies the relative influence of inertial forces compared to ST forces, which is crucial for predicting the behavior of bubbles and foams under various conditions. For instance, in processes like froth flotation in mineral processing or in the formulation of foams for industrial applications, understanding how bubbles and foams react to changes in inflow conditions and system constraints is essential. This numeral helps to forecast phenomena such as bubble coalescence or breakup and foam stability. This knowledge is vital for optimizing liquid systems because it provides detailed insights into how different forces affect the dynamics of multiphase mixtures. By leveraging this information, engineers and scientists can improve the efficiency and effectiveness of processes involving bubbles and foams, leading to enhanced performance in applications such as chemical reactors, separation technologies, and material processing.

### The case of the real coefficients

Hither, a particular case is deemed. In this case, Eq. (34) when $W_{e} \to 0$ may be expressed as:

35 $$\xi^{\prime \prime } + r_{1} \,\xi^{\prime } + r_{3} \,\xi + r_{5} \,\xi \xi^{\prime \prime } + r_{6} \,\xi \xi^{\prime } + r_{8} \,\,\xi^{2} \, + r_{10} \,\xi^{2} \xi^{\prime \prime } + r_{11} \,\xi^{\prime } \xi^{2} + r_{13} \,\xi^{3} = 0.$$

Thereby, the real portions are retained, whilst the imaginary ones will vanish.

**Fig. 2:**
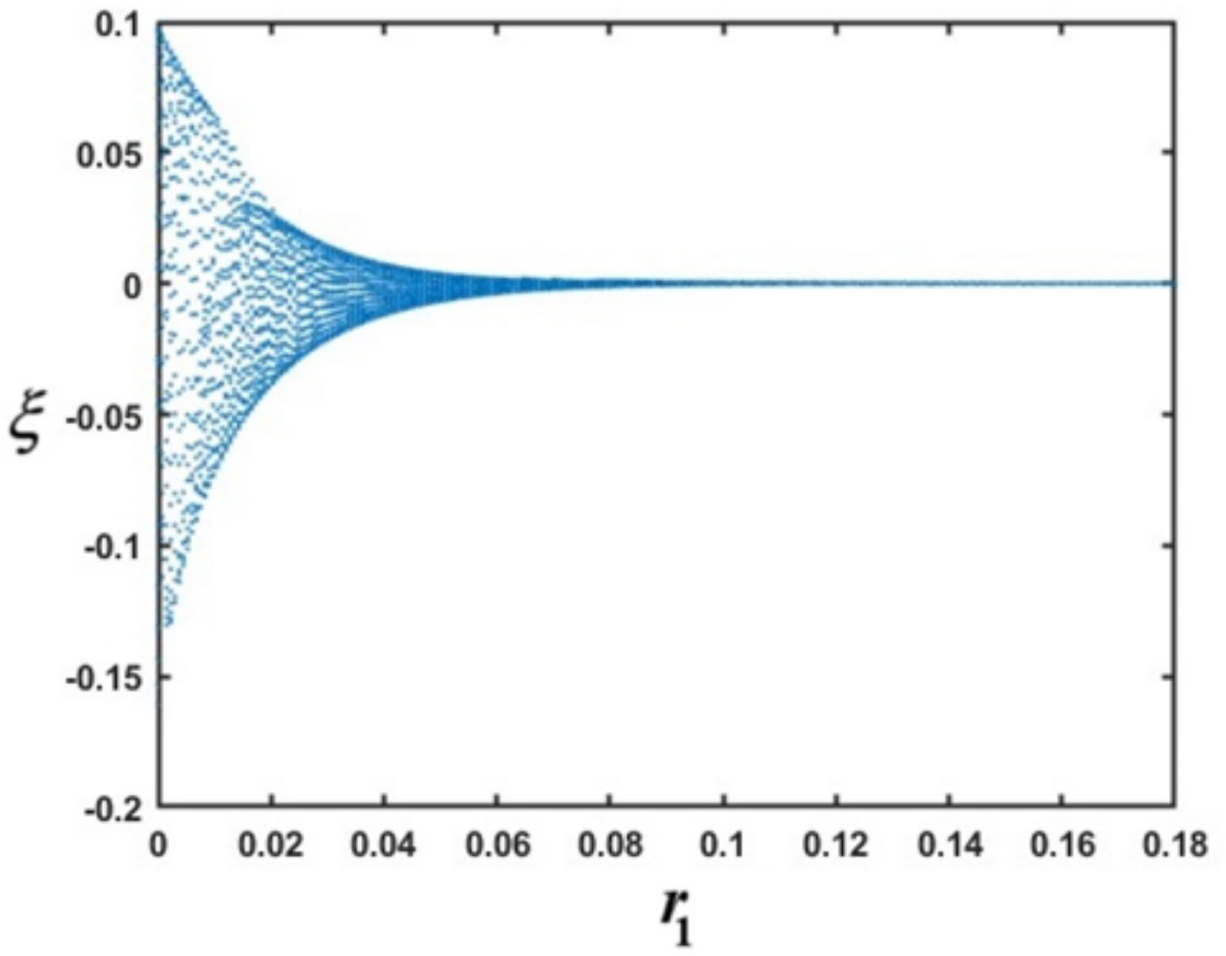
Elucidates bifurcation diagram for $\xi$ at $r_{3} = 0.02,\,r_{5} = 0.03,\,r_{6} = 0.04,\,r_{8} = 0.1,\,r_{10} = 0.2,\,r_{11} = 0.3,\,\,\,{\text{and}}\,\,\,r_{13} = 0.01$.

For more convenience, a bifurcation diagram of Eq. (35) will be plotted. As well known, bifurcation denotes a phenomenon in dynamical systems wherein a minor alteration in a system’s parameters precipitates a rapid qualitative transformation in its behavior. As the system’s control parameters fluctuate, it may shift from one state to another, such as from stability to instability or from periodic behavior to chaotic dynamics. This behavior is essential for comprehending the emergence of intricate phenomena across several disciplines, including physics, biology, and economics. A bifurcation diagram is utilized to illustrate these transitions, depicting the alterations in the system’s equilibrium points or periodic solutions as a function of the control parameter. Bifurcations elucidate the mechanisms behind abrupt or severe behavioral shifts in systems, frequently resulting in chaotic dynamics. Therefore, bifurcation is a change in system behavior when a certain parameter is changed that leads to a change in behavior from a stable state to unstable or vice versa. A comprehensive analysis of the bifurcation diagram was investigated in^12–14^ and^41–43^. It is noticed that in Fig. 2, in the region $r_{1} \in \left[ {0,\,0.08} \right]$ many points appear randomly which indicates that the motion behaves in chaotic behavior. In the second range, $\left( {0.08,\,0.18]} \right.$ we observed that a straight line, which means the motion, is stable.

The current study seeks to convert the formerly mentioned nonlinear ODE into a linear form by applying NPA. Following the methodology as formerly outlined^24–35^, the left-hand side of Eq. (35) is analyzed as consisting of three distinct components. Consequently, Eq. (35) can be reformulated to reflect this decomposition, resulting in a revised expression that facilitates further analysis and solution.

36 $$\xi^{\prime \prime } + G\left( {\xi ,\,\xi^{\prime } } \right) + M\left( {\xi ,\xi^{\prime } ,\xi^{\prime \prime } } \right) + F\left( {\xi \,,\xi^{\prime } ,\xi^{\prime \prime } } \right) = 0$$

and

37 $$\left. {\begin{array}{*{20}c} {G(\xi ,\xi^{\prime } ) = r_{1} \xi^{\prime } + r_{11} \,\xi^{2} \xi^{\prime } } \\ {M(\xi ,\xi^{\prime } ,\xi^{\prime \prime } ) = r_{5} \xi \xi^{\prime \prime } + r_{6} \,\xi \xi^{\prime } + r_{8} \,\xi^{2} } \\ {F(\xi ,\xi^{\prime } ,\xi^{\prime \prime } ) = r_{3} \,\xi + r_{10} \,\xi^{2} \xi^{\prime \prime } + r_{13} \,\xi^{3} } \\ \end{array} } \right\},$$

where $G(\xi ,\,\xi^{\prime } )$ is odd secular functions; $M(\xi ,\xi^{\prime } ,\xi^{\prime \prime } )$ refers to even non-secular functions, and $F(\xi ,\xi^{\prime } ,\xi^{\prime \prime } )$ stands for the odd secular functions.

The equivalent Linear ODE may be formulated as:

38 $$\ddot{u} + \Pi_{eqv} \dot{u} + \Theta_{eqv}^{2} \,\,u = \Lambda ,$$

with the initial conditions ICs:

39 $$u(0) = A,\,{\text{and}}\;u^{\prime } (0) = 0,$$

Subsequently, the guessing solution is presupposed to:

40 $$u(t) = A\cos \Omega t \Rightarrow u^{\prime}(t) = - A\Omega \sin \Omega t\& u^{\prime\prime}(t) = - \Omega^{2} u(t),$$

Following^24–35^, the three portions represented in Eq. (38) may be evaluated as:

41 $$\Theta_{eqv}^{2} = \int\limits_{0}^{2\pi /\Omega } {uF(u,\,u^{\prime},u^{\prime\prime})} dt/\int\limits_{0}^{2\pi /\Omega } {u^{2} \,dt} = r_{3} + \frac{3}{4}A^{2} \left( {r_{13} - \Omega^{2} r_{10} } \right),$$

42 $$\Pi_{eqv} = \int\limits_{0}^{2\pi /\Omega } {u^{\prime } \,G(u,\,u^{\prime } ,u^{\prime \prime } )} dt/\int\limits_{0}^{2\pi /\Omega } {u^{\prime 2} dt} = r_{1} + \frac{{A^{2} }}{4}r_{11} .$$

and

43 $$\left. {\Lambda = M(u,\,u^{\prime } ,u^{\prime \prime } } \right|_{{\left( {u \to \chi_{1} A,\,u^{\prime } \to \chi_{1} A\Omega ,u^{\prime \prime } \to \chi_{1} A\Omega^{2} } \right)}} = \frac{{A^{2} }}{4}\left( {\Omega^{2} r_{5} + \Omega \,r_{6} + r_{8} } \right).$$

The calculations of both Eq. (35) as well as Eq. (38) are calculated by utilizing MS. As well, the dimensionless numerals for this graph are taken as:

$$\begin{gathered} W_{e} = 0,A = 0.1,\rho = 0.02,\mu = 0.05,D_{a} = 0.05,k = 0.5,H_{0} = 0.1,Ha^{2} = 0.05, \hfill \\ B_{d} = {0}{\text{.85,U}} = {0}{\text{.05}} \cdot \mu_{e} = {0}.5 \, \;{\text{and}}\;Z = 0.01. \hfill \\ \end{gathered}$$

**Fig. 3:**
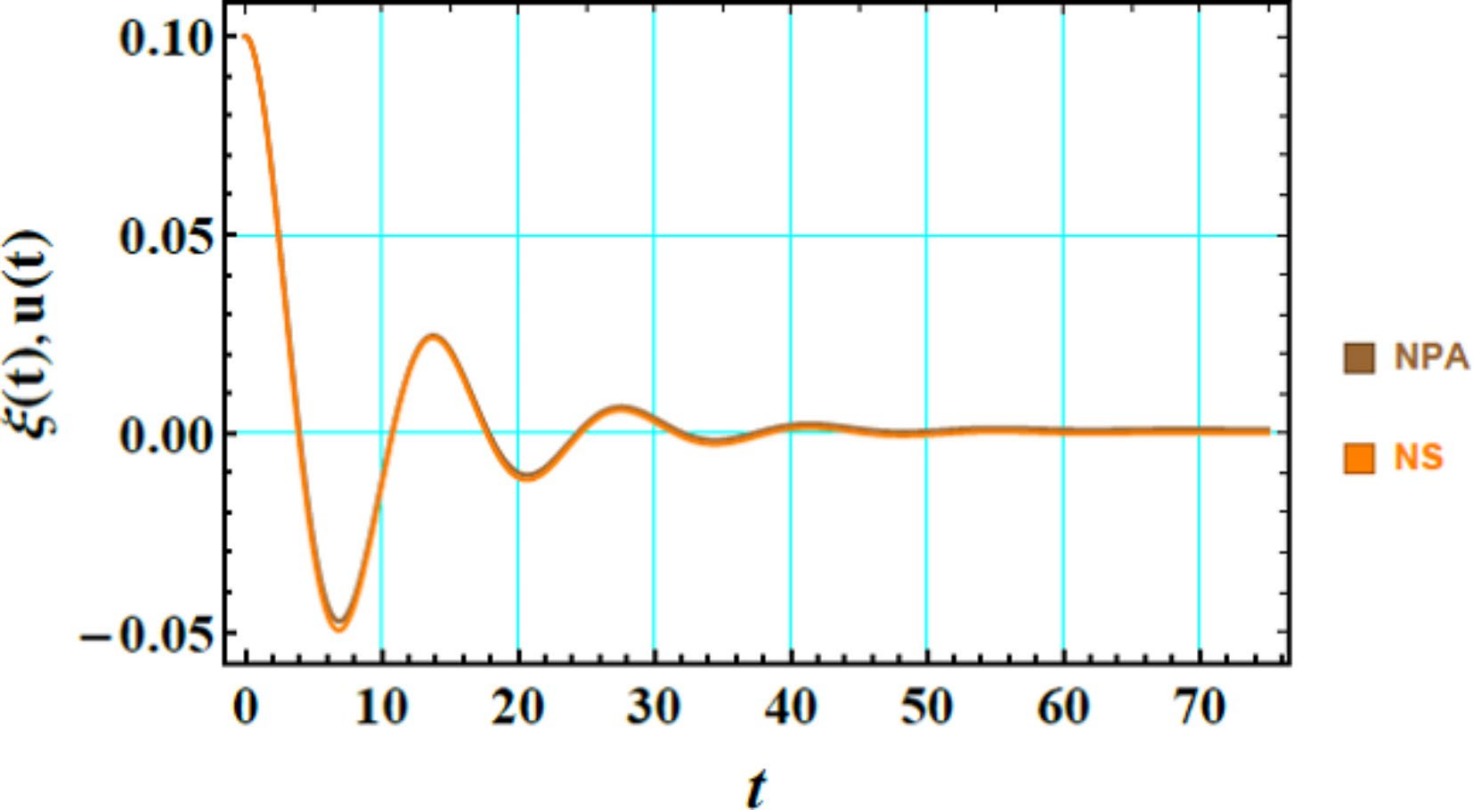
Compare the findings in Eqs. (35) and (38), respectively.

Fig. 3 illustrates the comparison between the NS of Eq. (35), represented by the orange curve, and the solution of Eq. (38), represented by the brown curve. The close alignment of these curves indicates a strong correlation between the two findings. The minimal discrepancy quantified as an error of merely 0.00279934, underscores the precision and reliability of the numerical method employed. This small error suggests that the numerical approach effectively captures the dynamics described by both equations, thus providing a robust and accurate representation of the problem at hand. Physically, this close agreement is crucial in validating the numerical procedures applied, indicating that the simulations are accurately modelling the physical phenomena described by the two equations. The low error margin reaffirms the effectiveness of the numerical techniques in solving complex differential equations, thereby enhancing confidence in the findings presented.

Table 1 provides error values comparing the linear and nonlinear results, showing very minor discrepancies between the two findings. These small differences suggest that the linear and nonlinear approaches are closely matched, indicating a strong agreement in the system’s conduct, regardless of which method is applied. From a physical perspective, this implies that nonlinear impacts are minimal, allowing the linear approximation to accurately reflect the system’s key dynamics. The slight variations emphasize the reliability and precision of both approaches, demonstrating that they yield nearly identical results with only marginal deviations.

**Table 1:** expounds error amounts that compare the linear and nonlinear outcomes.

Time	Real	Approximate	Absolute error
0	0.1	0.1	0
5	-0.0298656	-0.0270663	0.00279938
10	-0.0134309	-0.0123923	0.00103856
15	0.0204901	0.0212201	0.000730043
20	-0.0112353	-0.00994095	0.00129433
25	0.00139628	0.00224461	0.000848326
30	0.00287582	0.00385298	0.000977163
35	-0.00272573	-0.00170037	0.00102536
40	0.00108897	0.00202136	0.000932389
45	0.000121322	0.00111037	0.000989043
50	-0.000479506	0.000497644	0.00097715
55	0.000327903	0.00129329	0.000965387
60	-0.0000840092	0.00089535	0.000979359
65	-0.0000510568	0.000921354	0.000972411
70	0.0000691865	0.00104201	0.000972823
75	-0.0000354488	0.000939605	0.000975053

In light of Eq. (38), presuppose the standard normal form as:

44 $$u(t) = f(t)Exp( - \Pi_{eqv} t/2).$$

Accordingly,

45 $$\ddot{f}(t) + \left( {\Theta_{eqv}^{2} - \frac{1}{4}\Pi_{eqv}^{2} } \right)f(t) = - \Lambda Exp(\Pi_{eqv} \,t/2),$$

Consequently, the total frequency may be expressed as:$\Omega^{2} = \Theta_{eqv}^{2} - \frac{1}{4}\Pi_{eqv}^{2}$.

The stability criteria may be represented as:

46 $$\Omega^{2}> 0,\,{\text{and}}\;\,\Pi_{eqv}> 0$$

**Fig. 4:**
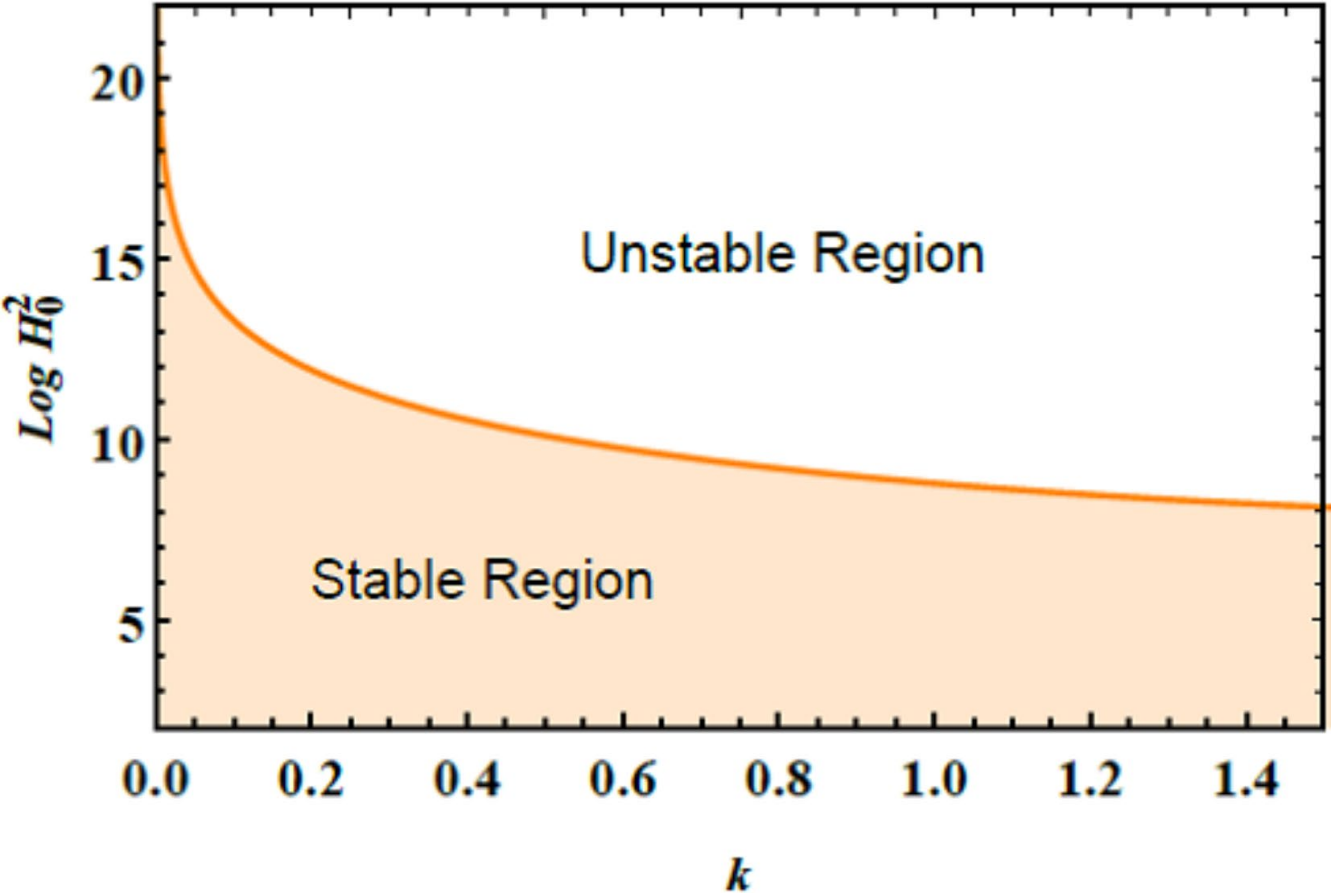
Clarifies the unstable & stable areas vs.$k$.

**Fig. 5:**
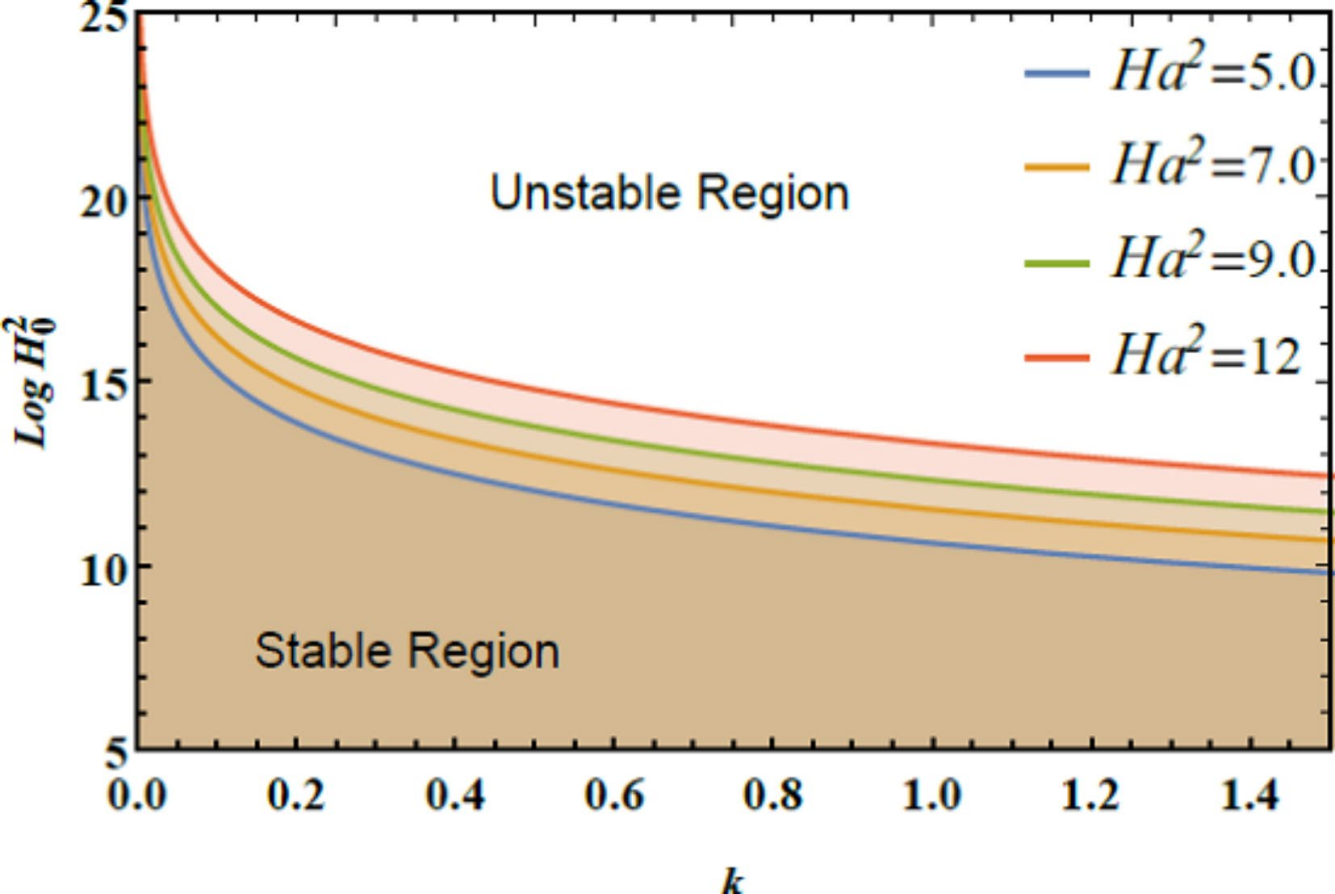
Displays $Ha^{2}$ on the stability area.

**Fig. 6:**
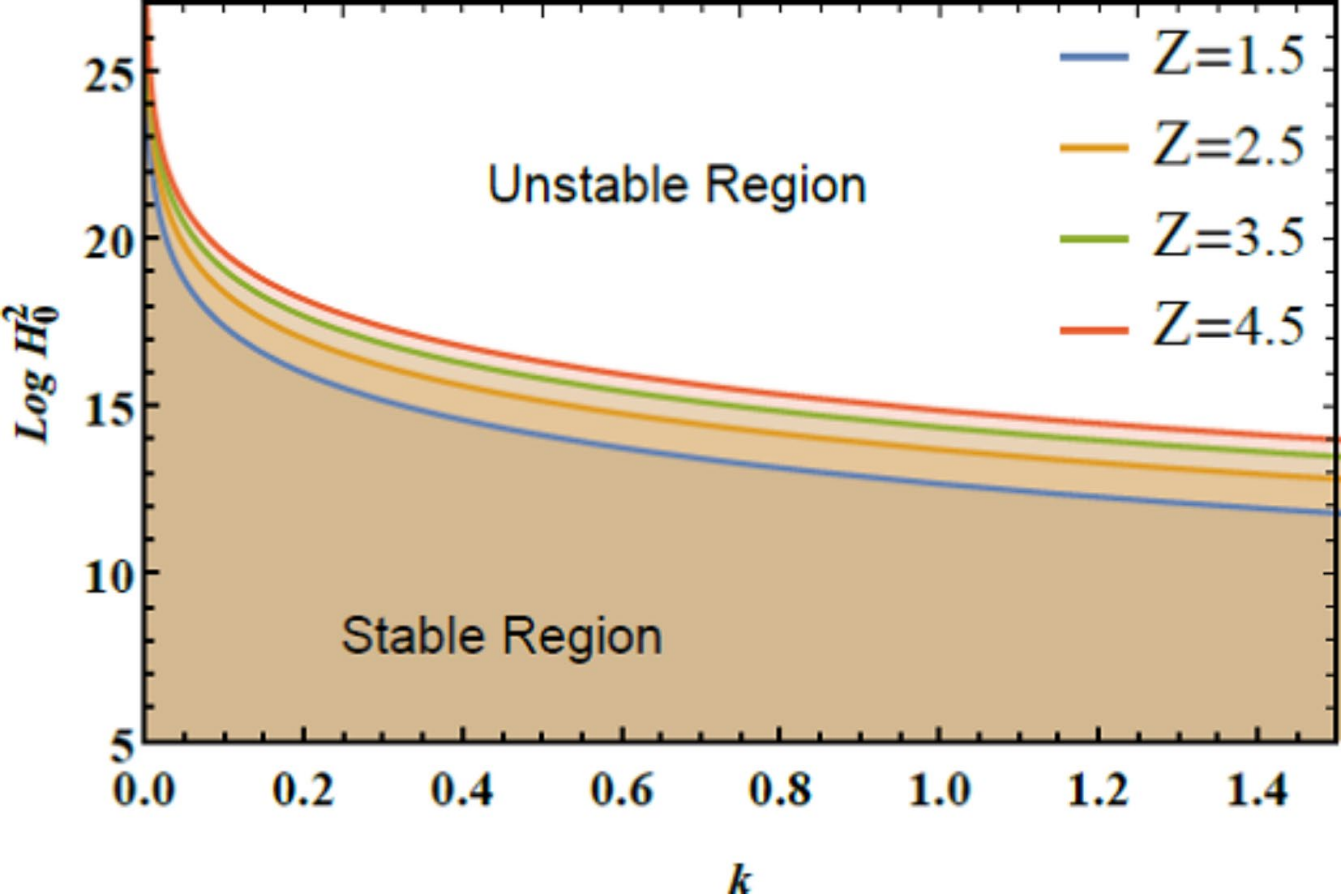
Expounds $Z$ on the stability diagram.

**Fig. 7:**
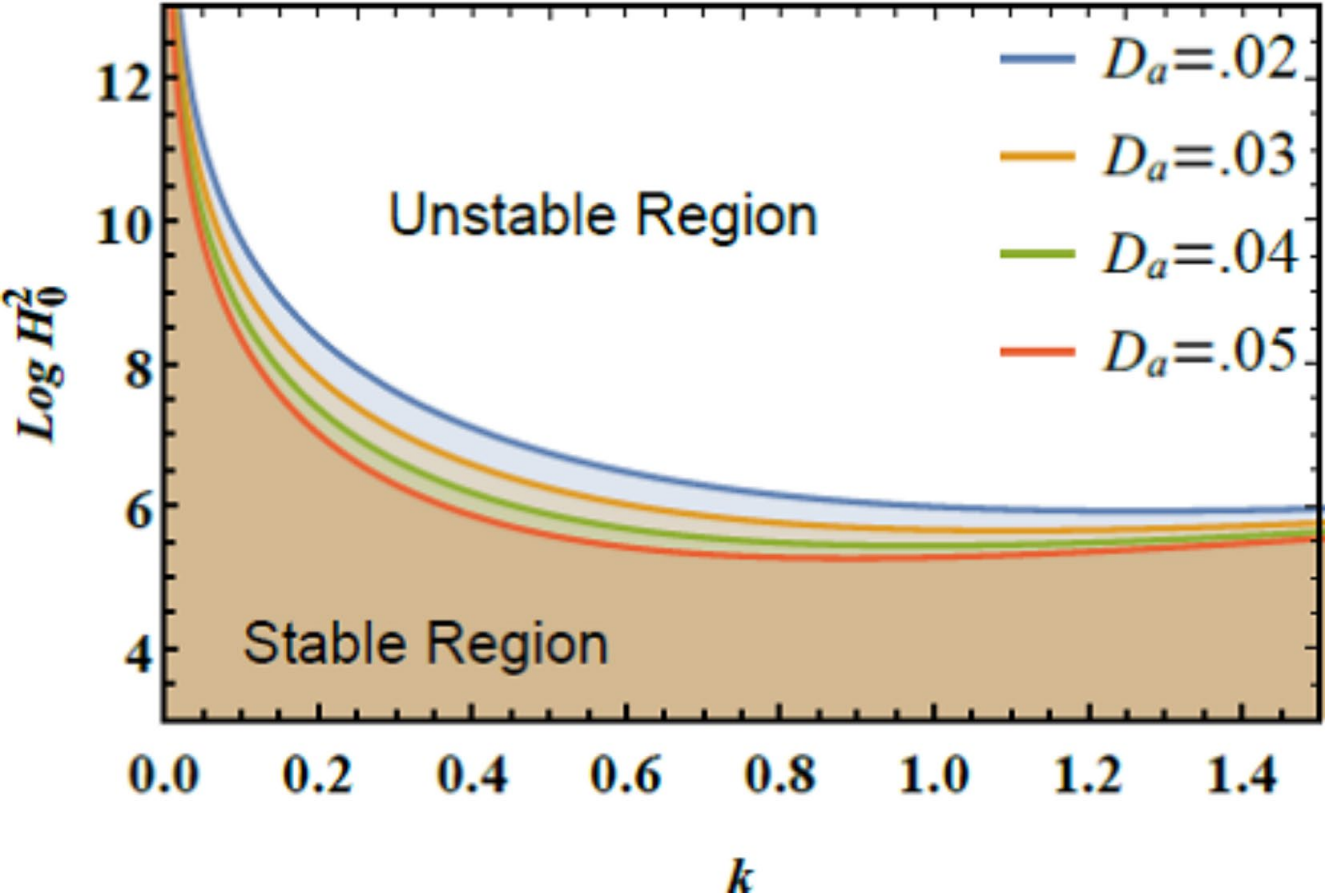
Demonstrates $D_{a}$ on the stability zone.

The stability graphs are sketched for $Log\,H_{0}^{2}$ vs. $k$ through Figs. 4, 5, 6, and 7. As well, the dimensionless numerals for these figures are chosen as:

$W_{e} = 0,\,A = 0.5,\,\rho = 0.02,\,\mu = 0.5,\,D_{a} = 0.05,\,Ha^{2} = 10,\,\Omega = 0.2B_{d} = 0.5,\,{\text{U}} = {0.9}.$ $\mu_{e} = 0.5 \,{\text{and}}\,Z = 1.5.$ Despite the factor $\Pi_{eqv}$ in the equivalent linear formula being independent of the MF, it is crucial to verify the restriction $\,\Pi_{eqv}> 0$ to ensure the completeness of the configuration. Considering this restriction, criteria (46) are expressed as $\alpha \,H_{0}^{2}> \delta$, where the constants $\alpha$ and $\delta$ are defined based on the details provided in the paper. Over and above, the numerical procedure has been demonstrated and validated $\alpha> 0$ for all $k> 0$, ensuring that the approach is comprehensive and accurate across the diverse scenarios regarded.

Fig. 4 elucidates the stability regions for $Log\,H_{0}^{2}$ vs.$k$. This figure demonstrates that the shaded region beneath the curve represents the stable zone, where the system operates in a consistent and steady state. Conversely, the area above the curve denotes the unstable zone, where the system may undergo deviations or fluctuations, indicating potential instability. Understanding this distinction is vital for various practical applications. For instance, liquid dynamics helps differentiate between stable and turbulent inflow restrictions in pipelines, ensuring efficient and predictable liquid transition. In engineering, identifying stable operating constraints is crucial for maintaining reliable and safe system performance. In the field of biology, recognizing stable regions is essential for modelling the spread of diseases and devising effective strategies for managing outbreaks and preventing further transmission.

Fig. 5 demonstrates how the stability diagram of $Log\,H_{0}^{2}$ vs. $k$ changes with varying amounts of the Hartmann numeral $Ha^{2}$. As depicted, the stable region of the diagram expands with increasing Hartmann number, indicating that the Hartmann numeral $Ha^{2}$ exerts a stabilizing impact on the system. Physically, the Hartmann numeral $Ha^{2}$ quantifies the relative strength of magnetic forces compared to viscous forces in a fluid. When the MF is applied perpendicular to the inflow direction, it generates a drag force known as the Lorentz force. This force acts to oppose fluid movement, From the physical perspective, influencing the flow conduct by enhancing the MF’s capacity to stabilize the inflow and reduce turbulence. In practical contexts, such as in metallurgical processes where molten metals are cooled or processed, the Hartmann numeral $Ha^{2}$ is crucial for optimizing the performance of MFs. By adjusting the MF strength to manage the Hartmann numeral, engineers can improve liquid stability, control turbulence, and enhance process efficiency.Similarly, in cooling systems for electronics, the Hartmann numeral plays a crucial role. It helps in designing systems that efficiently manage liquid performance under magnetic influences. This understanding enhances thermal management. As a result, the overall system performance improves significantly, including in applications like MHD power generation.^28^.

Fig. 6 illustrates the stability profile of $Log\,H_{0}^{2}$ vs. $k$ for diverse measures of the Ohnesorge numeral $Z$. This figure shows that as the Ohnesorge numeral $Z$ elevates, the stable region of the diagram expands. This indicates that the Ohnesorge numeral $Z$ exerts a stabilizing influence on the stability allocation. The Ohnesorge numeral $Z$ quantifies the ratio of viscous forces to ST forces in a liquid. Physically, a higher Ohnesorge numeral $\left( {Z> 1} \right)$ indicates that viscous forces dominate over ST forces. This performance has significant practical implications. In free-surface liquids, such as in reservoirs or open channels. A higher Ohnesorge numeral $\left( {Z> 1} \right)$ improves predictions of surface behavior, wave formation, and overall stability. At high Ohnesorge amounts, the dominance of viscous forces effectively dampens oscillations and suppresses instabilities, preventing phenomena such as droplet breakup, jet fragmentation, or interfacial wave development. The increased viscosity dissipates energy that would otherwise fuel disturbances. In fluid jets and films, ST can drive capillary instabilities like the Rayleigh-Plateau instability, which leads to droplet formation. A high Ohnesorge numeral counteracts these forces by balancing ST with viscous damping, stabilizing the system and preventing rapid deformation or fragmentation. In spray technology, including agricultural and industrial applications, it enhances droplet formation and atomization by escalating the influence of viscous forces. Thus, aiding in the control of droplet size and stability. In material processing, such as polymer and metal production. Adjusting the Ohnesorge numeral $Z$ allows for better management of liquid dynamics, resulting in improved product quality. This observation aligns with former research^28^, which confirmed the stabilizing impact of the Ohnesorge numeral $Z$ on liquid stability, reinforcing its importance in maintaining consistent and reliable fluid conduct in various contexts.

Fig. 7 shows the stability profile of $Log\,H_{0}^{2}$ vs. $k$ for diverse amounts of the Darcy numeral $D_{a}$. It shows that escalating the Darcy numeral $D_{a}$ causes a destabilization of the system, with the stability zone shrinking as the Darcy numeral $D_{a}$ rises. This indicates that a higher Darcy numeral leads to reduced stability in the system. Physically, the Darcy numeral $D_{a}$ quantifies the ratio of liquid permeability to the product of liquid viscosity and a characteristic length scale. It reflects how easily fluid can flow through a porous medium relative to the resistance offered by the medium’s internal structure. A higher Darcy numeral implies higher permeability and, consequently, a lower resistance to fluid inflow. When the Darcy numeral enlarges, the porous medium allows more liquid to pass through with less resistance. This reduction in resistance can lead to a more turbulent flow or less controlled fluid dynamics, which destabilizes the system. Essentially, the system becomes less stable because the increased fluid movement through the porous structure disrupts the existing flow patterns, potentially leading to chaotic or unpredictable behavior. The Darcy numeral is crucial across several fields. In biological sciences, it affects how liquids move through tissues and scaffolds, impacting nutrient and drug delivery. Technology influences the performance of filtration and catalytic systems, where a high Darcy number can lead to unstable flow and lower efficiency. In Medicine, it is the key to designing medical devices that work with porous media, such as drug delivery systems and tissue implants. In petroleum engineering, Darcy’s law, which uses the Darcy numeral, describes liquid movement in reservoirs, with higher measures indicating greater fluid inflow through rocks, influencing extraction and reservoir management. These applications underscore the significance of managing liquid permeability to ensure stability and optimal performance in diverse systems, aligning with former research findings^28^.

**Fig. 8:**
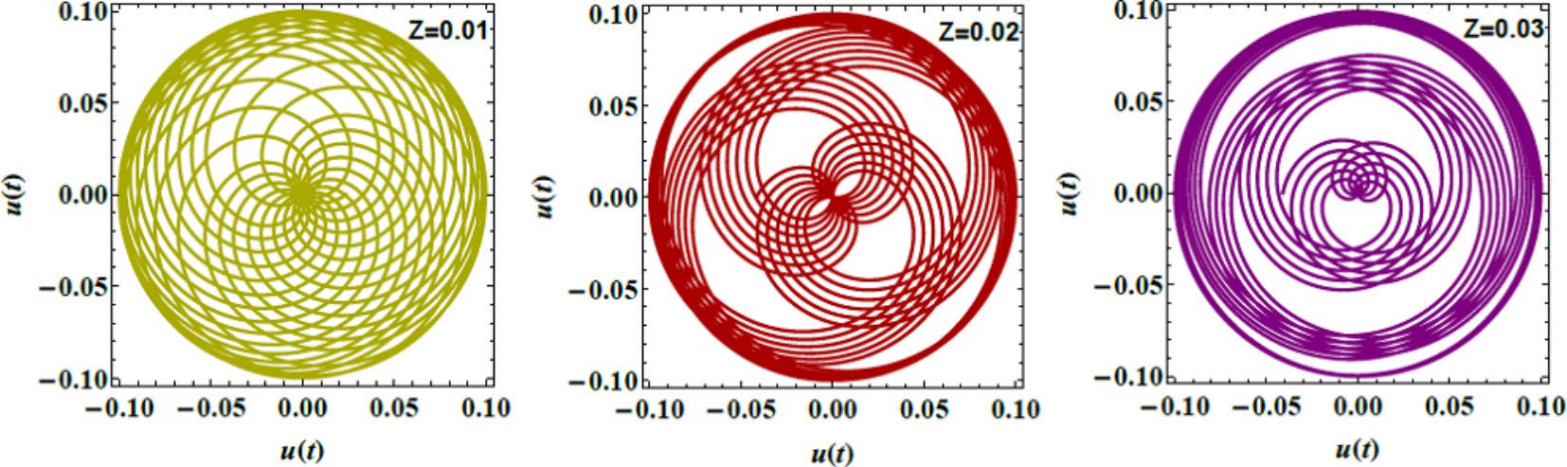
Demonstrates $$u(t)$$ for $$Z$$.

**Fig. 9:**
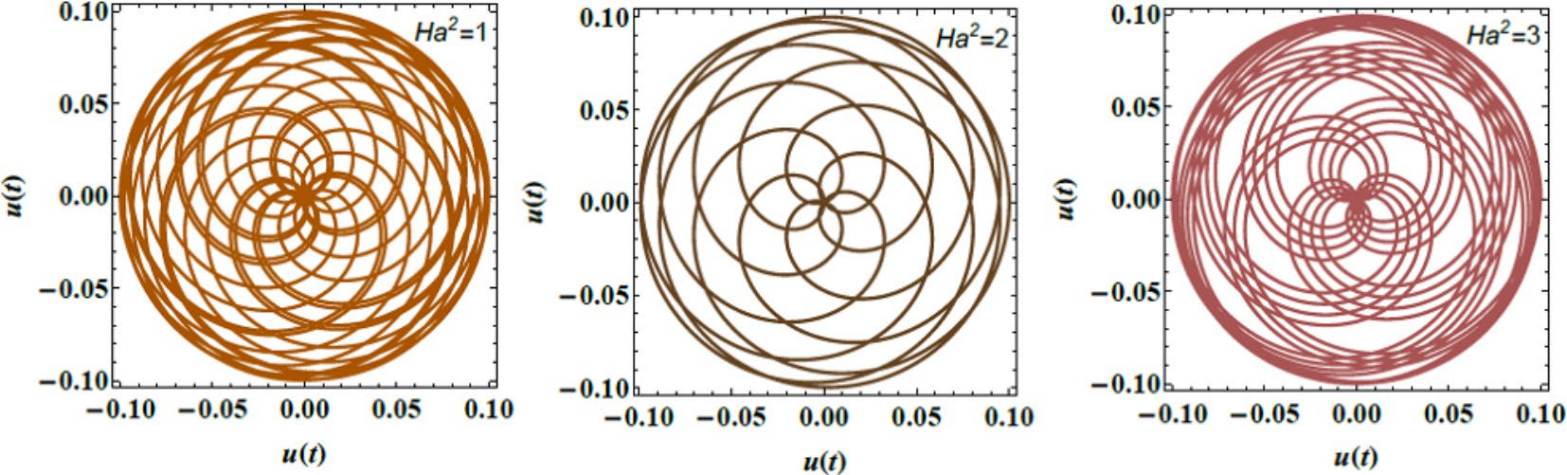
Expounds $$u(t)$$ for $$Ha^{2}$$ .

**Fig. 10:**
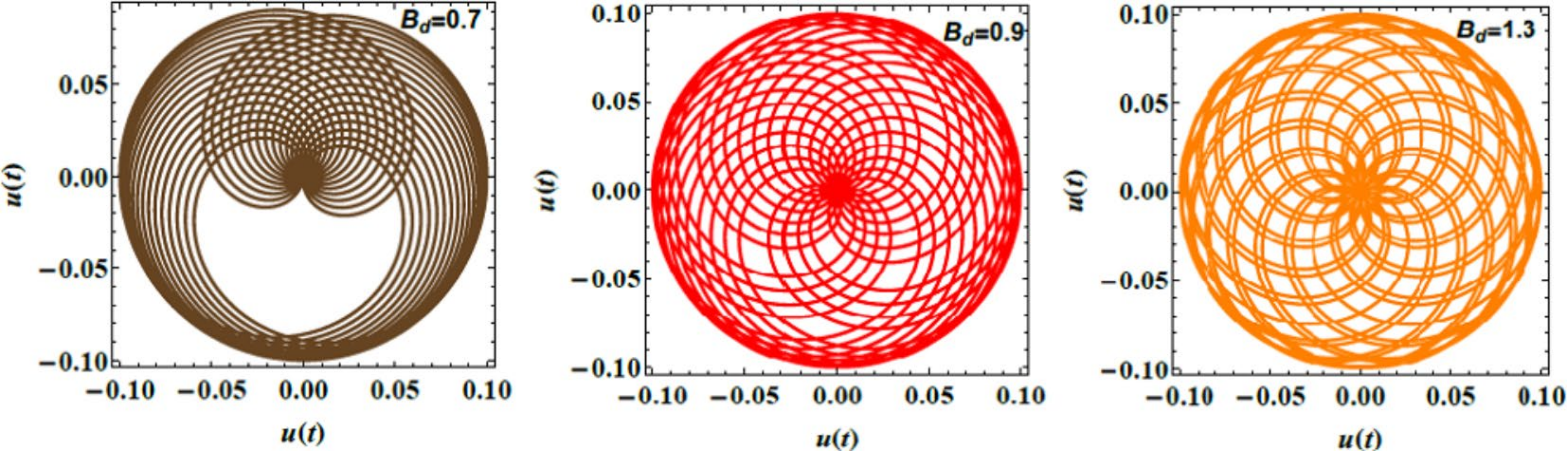
Elucidates $$u(t)$$ for $$B_{d}$$ .

Figs. 8, 9, and 10 demonstrate the NS for the function $u(t)$ that is plotted against time applying the PolarPlot command from Eq. (40). The variations in the Ohnesorge numeral $Z$, the Hartmann numeral $Ha^{2}$, and the Bond numeral $B_{d}$ are regarded. These figures show that the curves exhibit symmetrical patterns around their centers. These centers shifted based on changes in the Ohnesorge, Hartmann, and Bond numerals. As these numerals escalate, they influence the curves’ symmetry and position, reflecting changes in the liquid dynamics described by these parameters. The numeral of oscillations either escalates or dwindles depending on changes in these parameters. Specifically, variations in the Ohnesorge, Hartmann, and Bond numerals affect the frequency and amplitude of oscillations recognized in the liquid system. Physically, the Ohnesorge numeral quantifies the ratio of viscous forces to ST forces in a liquid. This affects how these forces balance out. Changes in the Ohnesorge numeral modify the fluid’s performance, impacting droplet formation and stability in systems. This occurs as ST and viscosity play significant roles. The Hartmann numeral measures the relative strength of MFs compared to viscous forces. This numeral influences how MFs stabilize or destabilize liquid inflow. As the Hartmann numeral enhances, it reflects the impact of magnetic forces on liquid dynamics. This is crucial in implementations like MF-assisted liquid processing and cooling systems. The Bond numeral evaluates the relative significance of gravitational forces compared to ST. It helps determine the stability of liquid interfaces and the shape of droplets or bubbles. Variations in the Bond numeral affect how gravity influences liquid conduct, which is vital in processes such as fluid inflow in porous media and the formation of liquid layers. These variations are crucial for implementations such as liquid inflow analysis in engineering, spray technology, and material processing. Understanding how these numerals affect the stability and performance of liquid systems aids in optimizing performance and predicting system responses under diverse constraints^28,31,32,34^.

## Stability analysis of the complex situation

In this case, the Weber numeral $We$ is deemed. Supplementary, the preceding procedures that are applied in Eq. (35) will be applied here. For simplicity, Eq. (34) may be represented as:

47 $$\xi^{\prime \prime } + \eta \xi^{\prime } + \Gamma \xi + r_{5} \xi \xi^{\prime \prime } + R\xi \xi^{\prime } + Q\xi^{2} + r_{10} \xi^{2} \xi^{\prime \prime } + S\xi^{\prime } \xi^{2} + K\xi^{3} = 0,$$

where the dash signifies time-derivative, all factors are complex and may be expressed as:

48 $$\eta = (r_{1} + ir_{2} ),\;\Gamma = (r_{3} + ir_{4} ),\;R = (r_{6} + ir_{7} ),\;Q = (r_{8} + ir_{9} ),\;S = (r_{11} + ir_{12} )\;{\text{and}}\;K = (r_{13} + ir_{14} ).$$

The NPA is relying on getting the equivalent formula to Eq. (47). Consequently, Eq. (47) can be introduced as:

49 $$\xi^{\prime \prime } + E(\xi ,\xi^{\prime } ) + D(\xi ,\xi^{\prime } ,\xi^{\prime \prime } ) = - N(\xi ,\xi^{\prime } ,\xi^{\prime \prime } ),$$

where $E(\xi ,\xi^{\prime } )$ is the damping odd functions, $D(\xi ,\xi^{\prime } ,\xi^{\prime \prime } )$ is the secular odd components, and $N(\xi ,\xi^{\prime } ,\xi^{\prime \prime } )$ is the non-secular quadratic components. They are given as:

50 $$\left. {\begin{array}{*{20}c} {E(\xi ,\xi^{\prime } ) = \eta \xi^{\prime \prime } + S\xi^{\prime } \xi^{2} } \\ {D(\xi ,\xi^{\prime } ,\xi^{\prime \prime } ) = \Gamma \xi + r_{10} \xi^{2} \xi^{\prime \prime } + K\xi^{3} } \\ {{\text{and}}\;N(\xi ,\xi^{\prime } ,\xi^{\prime \prime } ) = r_{5} \xi \xi^{\prime \prime } + R\xi \xi^{\prime } + Q\xi^{2} } \\ \end{array} } \right\}.$$

The primary trial solution is supposed as in the former case^24–35^:

51 $$q = B\cos \Delta t,$$

where $\Delta$ is the total frequency.

As well, the ICs are: $q(0) = B,\,\,$ and $q^{\prime}\left( 0 \right) = 0$. Thereby, one gets

52 $$q^{\prime \prime } + {\rm X}_{eqv} q^{\prime } + \chi_{eqv}^{2} q = - \varpi .$$

As in the former case, one grows

53 $${\rm X}_{eqv} = \int\limits_{0}^{2\pi /\Delta } {q^{\prime } \,E(q,\,q^{\prime } )} dt/\int\limits_{0}^{2\pi /\Delta } {q^{\prime 2} \,dt} = \eta + \frac{{B^{2} }}{4}S,$$

54 $$\chi_{eqv}^{2} = \int\limits_{0}^{2\pi /\Delta } {q\,D(q,\,q^{\prime } ,q^{\prime \prime } )} dt/\int\limits_{0}^{2\pi /\Delta } {q^{2} \,dt} = \Gamma + \frac{3}{4}B^{2} \left( {K - \Delta^{2} r_{10} } \right),$$

and

55 $$\varpi = \left. {N(q,\,q^{\prime } ,q^{\prime \prime } )} \right|_{{\,\,\left( {q \to \frac{B}{2},\,q^{\prime } \to \frac{B\Delta }{2},\,q^{\prime \prime } \to \frac{{B\Delta^{2} }}{2}} \right)}} = \frac{{B^{2} }}{4}\left( {\Delta^{2} \,r_{5} + \Delta \,R + Q} \right),$$

Equations (52-55) can be transformed into its normal formula as:

56 $$q(t) = \gamma^{*} (t)\,Exp( - {\rm X}_{eqv} t/2).$$

Consequently, $$\gamma^{*} (t)$$ verifies:

57 $$\gamma^{*^{\prime\prime}} (t) + \Delta^{2} \,\gamma^{*} (t) = - \varpi \,Exp({\rm X}_{eqv} t/2),\;\Delta^{2} = \left( {\chi_{eqv}^{2} - \frac{{{\rm X}_{eqv}^{2} }}{4}} \right)$$

The findings of Eq. (57) can be expressed as $\gamma^{*} (t) = \left. {\left. {\gamma^{*} } \right|_{C.f.} + \gamma^{*} } \right|_{P.I.}$, where

58 $$\left. {\gamma^{*} } \right|_{C.f.} = Exp(\Sigma + i\vartheta )t$$

and

59 $$\left. {\gamma^{*} } \right|_{P.I.} = - \frac{\varpi }{{\chi_{eqv}^{2} }}Exp({\rm X}_{eqv} t/2),$$

where $\Sigma$ and $\vartheta$ are real amounts.

Substituting from Eq. (58) in the homogenous portion of Eq. (57), one obtains:

60 $$(\Sigma + i\vartheta )^{2} + \Delta^{2} = 0,$$

So, the real and imaginary parts may be presented as follows:

61 $$4(\Sigma^{2} - \vartheta^{2} ) - r_{1}^{2} + r_{2}^{2} + 4r_{3} + 3B^{2} r_{13} - 3B^{2} \vartheta^{2} r_{10} - \frac{1}{2}B^{2} r_{1} r_{11} - \frac{1}{16}B^{4} r_{11}^{2} + \frac{1}{2}B^{2} r_{2} r_{12} + \frac{1}{16}B^{4} r_{12}^{2} = 0,$$

and

62 $$2\Sigma \vartheta - \frac{1}{2}r_{1} r_{2} + r_{4} + \frac{3}{4}B^{2} r_{14} - \frac{1}{8}B^{2} r_{2} r_{11} - \frac{1}{8}B^{2} r_{1} r_{12} - \frac{1}{32}B^{4} r_{13} r_{14} = 0.$$

Where the amounts of $\Sigma ,$ and $\vartheta$ are gained with the help of MS in addition, they are averted here to diminish the length of the article.

Subsequently, the whole outcome of Eq. (56) may be displayed as:

63 $$q(t) = Exp\left( {(\Sigma + i\vartheta ) - \frac{{{\rm X}_{eqv} }}{2}} \right)t - \frac{\varpi }{{\chi_{eqv}^{2} }} = q_{R} + i\,q_{I} .$$

Applying the preceding Eqs. (53) - (58) with (59), consequently, $q_{R}$ and $q_{I}$ may be computed as follows:

64 $$q_{R} = Exp(\Sigma - L_{1} /2)\,t\,\cos (\vartheta - L_{2} /2)\,t + (L_{3} L_{5} + L_{4} L_{6} )/(L_{3}^{2} + L_{4}^{2} ),$$

and

65 $$q_{I} = Exp(\Sigma - L_{1} /2)\,t\,{\text{Sin}} (\vartheta - L_{2} /2)\,t + (L_{3} L_{6} - L_{4} L_{5} )/(L_{3}^{2} + L_{4}^{2} ).$$

The factors $L_{1} \to L_{6}$ are moved to the Appendix to follow the paper easily.

The stability restriction may be demonstrated as:

66 $$\Sigma - L_{1} /2 < 0,$$

Tables 2 and 3 outline the stability constraints for diverse measures of the Hartmann and Weber numerals $Ha^{2}$. The outcomes demonstrate that these constraints hold across all tested amounts. This remains true even when the Weber numeral exceeds 1 $\left( {W_{e}> 1} \right)$. This consistency indicates that the stability criteria remain valid, regardless of the magnitude of the Hartmann and Weber numerals. Physically, this suggests that even when inertial forces dominate, the interaction between magnetic forces (represented by the Hartmann numeral) and ST effects (reflected in the Weber numeral) still adheres to the predicted stability limits. These findings offer key insights into liquid stability under varying magnetic and inertial forces, helping predict stability in practical applications such as MHD systems, liquid metal flows, and other fluid systems where both MFs and ST play crucial roles in determining performance.

**Table 2:** Display the amounts of $\Sigma - L_{1} /2 < 0$ for diverse amounts of $Ha^{2}$.

$Ha^{2}$	${\text{Re}} \left( {\Sigma - L_{1} /2} \right)$
1	-0.0572792
2	-0.0824976
3	-0.119637
4	-0.145558
5	-0.115851
6	-0.0846015
7	-0.0643308
8	-0.0506029
9	-0.0408475
10	-0.0336562

**Table 3:** Demonstrate the amounts of $\Sigma - L_{1} /2 < 0$ for district amounts of $W_{e}$.

$W_{e}$	${\text{Re}} \left( {\Sigma - L_{1} /2} \right)$
5	-0.0601277
10	-0.0485866
15	-0.0420026
20	-0.0376237
25	-0.0344649
30	-0.0320645
35	-0.0301722
40	-0.028639
45	-0.0273697
50	-0.0263006

**Fig. 11:**
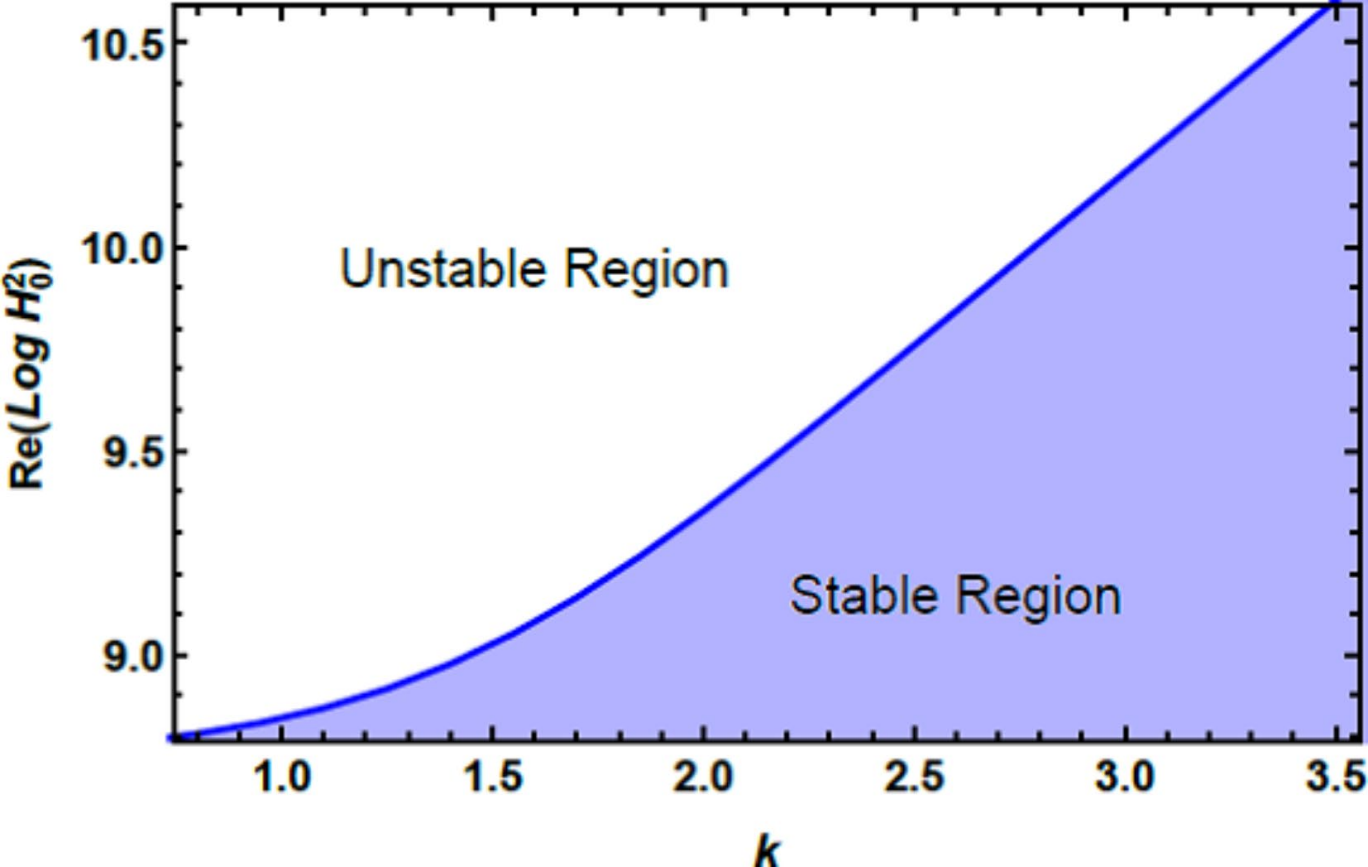
Describes the unstable & stable zones vs.$k$.

**Fig. 12:**
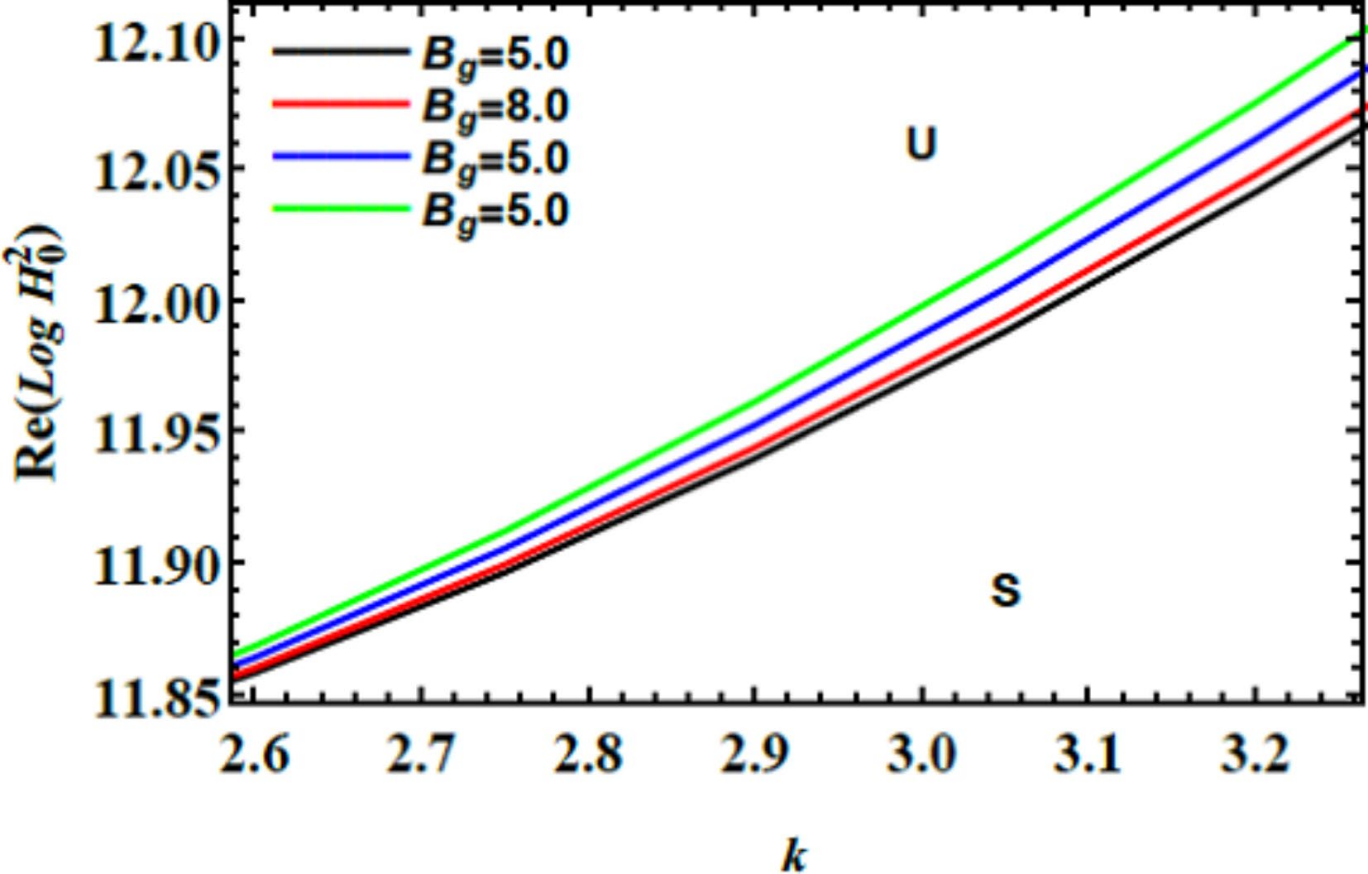
Clarifies $B_{g}$ on the stability area.

**Fig. 13:**
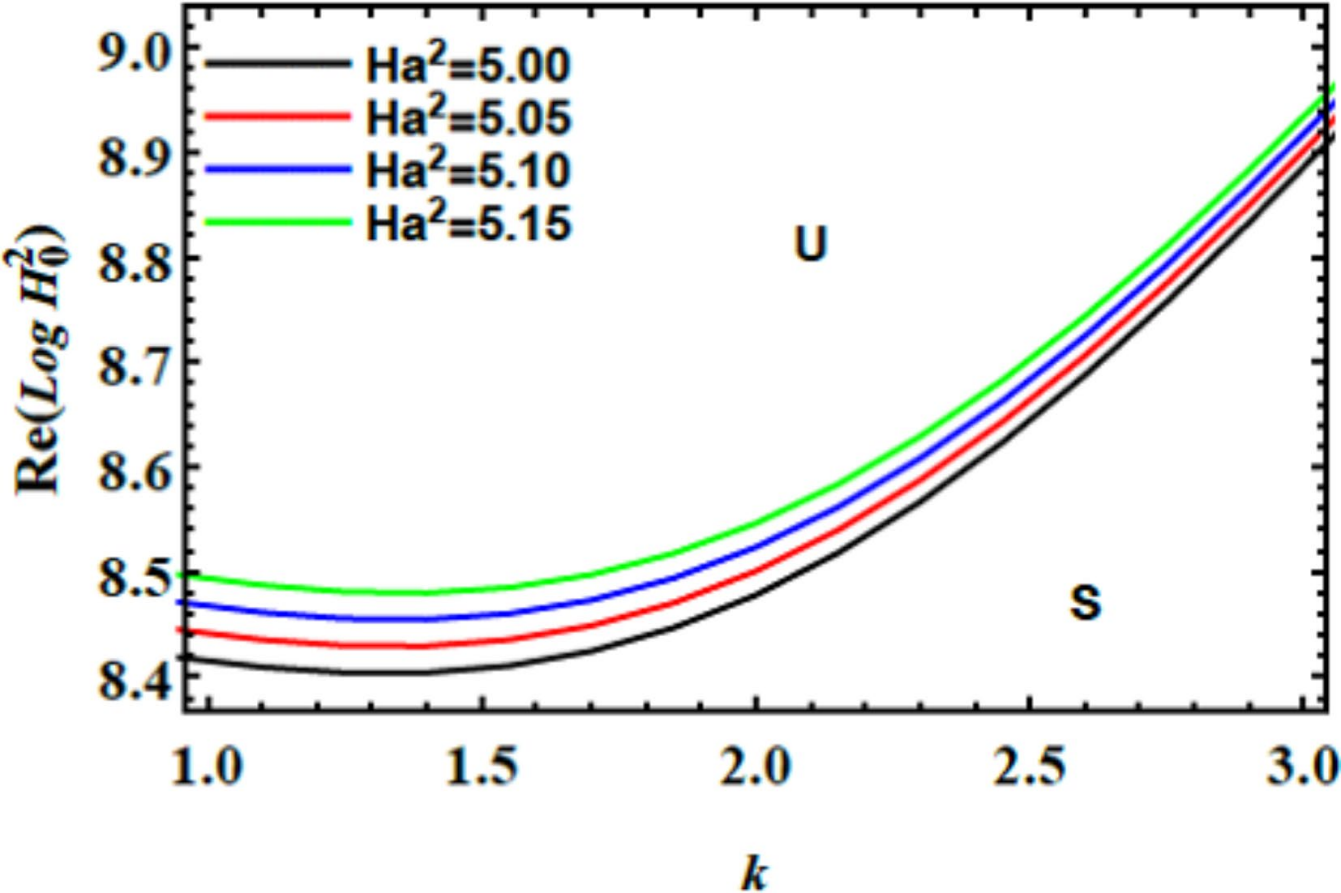
Explains $Ha^{2}$ on the stability area.

**Fig. 14:**
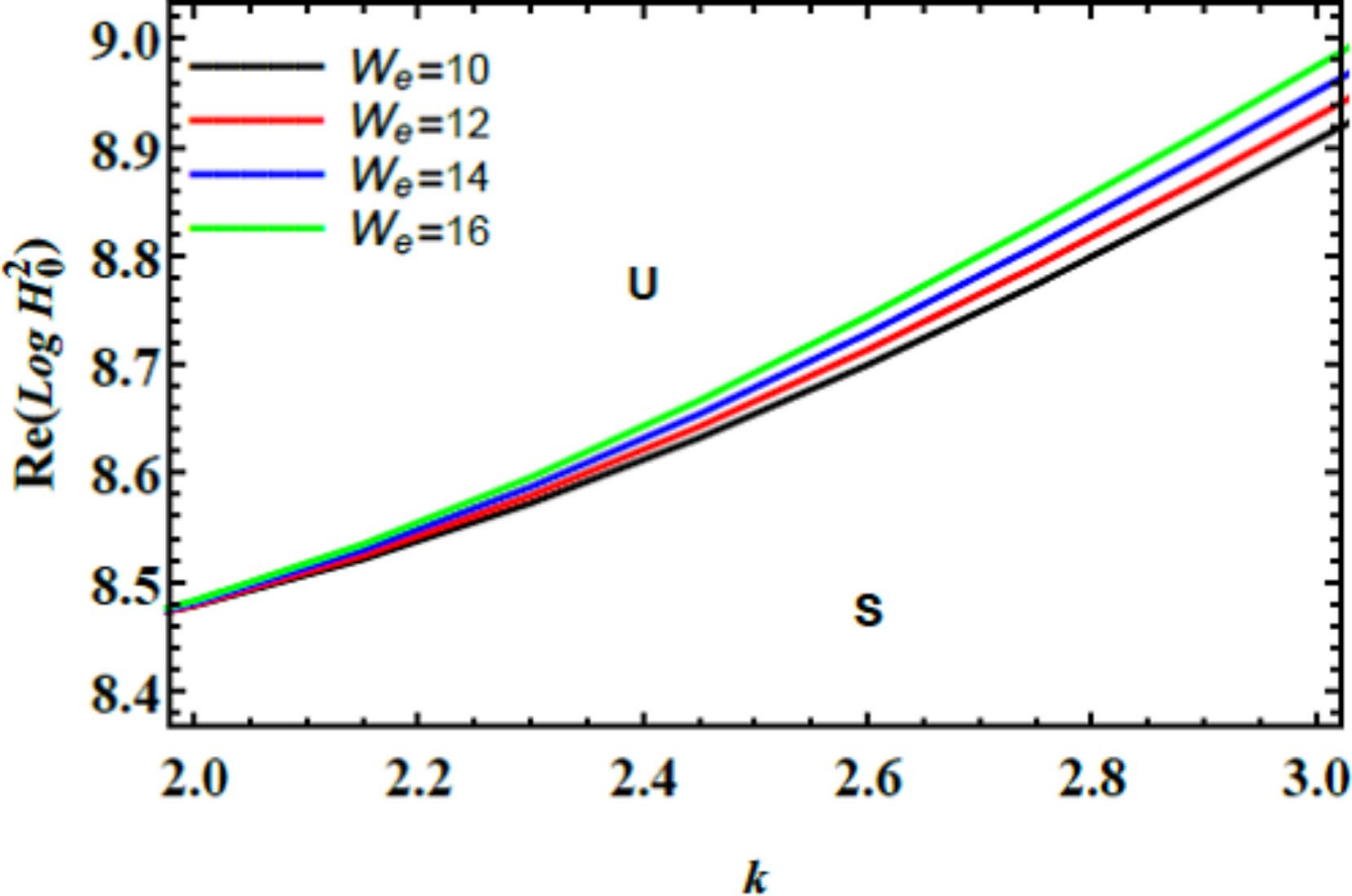
Displays $W_{e}$ on the stability area.

The equality described by criterion (66) forms a transcendental equation, which is inherently complex and challenging to solve directly. To facilitate a better understanding of the stability distribution, it is useful to visualize the relationship graphically. Thus, Figs. 11, 12, 13, and 14 illustrate how the MF numeral $${\text{Re}} \left( {Log\,H_{0}^{2} } \right)$$ varies with the wave number $$k$$. These graphs depict the real component of the MF numeral $${\text{Re}} \left( {Log\,H_{0}^{2} } \right)$$ as a function of the wave number $$k$$, providing a clearer view of its performance across distinct scenarios. The complexity of the MF numeral $${\text{Re}} \left( {Log\,H_{0}^{2} } \right)$$ values arises primarily from the presence of multiple square roots in the derived equation, leading to both real and imaginary components. However, for practical purposes, the figures focus on the real portion of the MF, which is more directly relevant to physical interpretations and applications. This approach allows for a more straightforward analysis of how different numerical amounts affect the stability of the system, offering valuable insights into the dynamics governed by the MF numeral and wave numeral. Therefore, the figures focus on plotting $${\text{Re}} \left( {Log\,H_{0}^{2} } \right)$$ vs. $$k$$ as a function of all: relevant parameters, providing a clearer depiction of the stability conduct as: $$W_{e} = 50,\,\,B = 0.5,\,\,\rho = 1,\,\,\mu = 0.3,\,\,\,D_{a} = 0.05,\,\,\,Ha^{2} = 10,$$$$\mu_{e} = {0}.5,$$ $$B_{d} = {1,}$$ $$\,B_{g} = 2.5{,}\,\,{\text{U}} = {0}{\text{.5}}$$$$\,{\text{and}}\,\,\,\,Z = 5.$$

Fig. 11 describes the stability diagram showing the relationship between the MF numeral ${\text{Re}} \left( {Log\,H_{0}^{2} } \right)$ and wave numeral $k$. In this diagram, the colored region below the curve represents the stable zone, where the system remains steady. Conversely, the lighter area above the curve indicates the unstable zone, where the system is prone to instability. This diagram is crucial for understanding how changes in ${\text{Re}} \left( {Log\,H_{0}^{2} } \right)$ and $k$ impact overall system stability. Stability diagrams like this have wide-ranging applications: in engineering, they aid in designing materials and structures by identifying stable parameter ranges. In liquid dynamics, they assist in predicting the stability of fluid flows in systems such as pipelines or reactors; in materials science. They help determine restrictions for material stability and phase transitions. Further, in biological systems, they guide the design of medical devices and treatments by pinpointing stable operating constraints. Overall, Fig. 11 offers valuable insights into stability and instability zones, with important implications across diverse fields^28,31,32,34^.

Fig. 12 depicts the variance of ${\text{Re}} \left( {Log\,H_{0}^{2} } \right)$ vs. $k$ for various amounts of the Bingham factor $B_{g}$. The diagram shows that as the Bingham factor $B_{g}$ enlarges, the stable region expands, indicating a stronger stabilizing influence on the system’s stability profile. This stabilization occurs due to the enlarged yield stress associated with Bingham plastic conduct. That resists inflow until a certain critical stress is reached. Physically, this means that a higher Bingham factor enhances the system’s resistance to instability. That makes it less prone to disturbances. This effect is crucial in applications such as rheology and liquid mechanics, where controlling the flow of non-Newtonian liquids like drilling muds or food pastes is essential. In material science, it helps in designing materials that maintain their integrity under stress, and in civil engineering. It aids in selecting materials for tasks like soil stabilization or concrete mixtures to ensure structural stability. Overall, this figure underscores the significant role of the Bingham factor in promoting stability, with broad implications across engineering and industrial fields^39,40^.

Fig. 13 describes the stability profile of ${\text{Re}} \left( {Log\,H_{0}^{2} } \right)$ vs. $k$ for varying amounts of the Hartmann numeral $Ha^{2}$. The diagram shows that as the Hartmann numeral enriches, the stable region on the plot expands, indicating that the Hartmann numeral exerts a stabilizing influence on the system’s stability profile. Physically, this stabilization occurs because the Hartmann numeral quantifies the ratio of magnetic forces to viscous forces in the liquid. As the MF strength increases, the Lorentz force, which acts perpendicular to the flow direction, becomes more pronounced. This force creates a drag impact that counteracts the fluid’s movement. That enhances stability by reducing disturbances and fluctuations. The expanding stable region reflects that stronger MFs can better control and stabilize liquid inflows. That causes more predictable and controlled performance in systems where MFs influence liquid dynamics. This stabilizing impact has significant implementations: in engineering, it can improve the design of magnetic liquid systems and reactors. Additionally, in material science, it aids in controlling processes involving MFs. Further, in industrial implementations, it enhances the stability of liquids subjected to magnetic forces, such as in magnetic separation and processing technologies^28,31,32,37,38^.

Fig. 14 depicts how the stability profile of ${\text{Re}} \left( {Log\,H_{0}^{2} } \right)$ vs. $k$ changes with varying amounts of the Weber numeral $\left( {W_{e}>> 1} \right)$. This figure shows that as the Weber numeral $\left( {W_{e}>> 1} \right)$ escalates, the system’s stability improves, indicating a stabilizing influence. This occurs because the Weber numeral quantifies the ratio of inertial forces to ST forces. As the Weber numeral rises, inertial forces become more dominant compared to ST. That leads to a more stable liquid interface. Physiologically, this means that the liquid system becomes less sensitive to fluctuations in ST. Thereby reducing instability. This stabilizing impact has practical implementations in several fields. In material processing, it can enhance the uniformity of sprays or coatings. In addition, in chemical engineering, it improves mixing and reaction stability. Further, in aerospace, it optimizes fuel atomization. Finally, in biological systems, it assists in predicting and controlling liquid conduct in suspensions. Overall, the enhancing Weber numeral contributes to greater system stability by shifting the balance towards inertial forces, which can be crucial for optimizing performance and stability in diverse industrial and scientific implementations^28,31,32^.

**Fig. 15:**
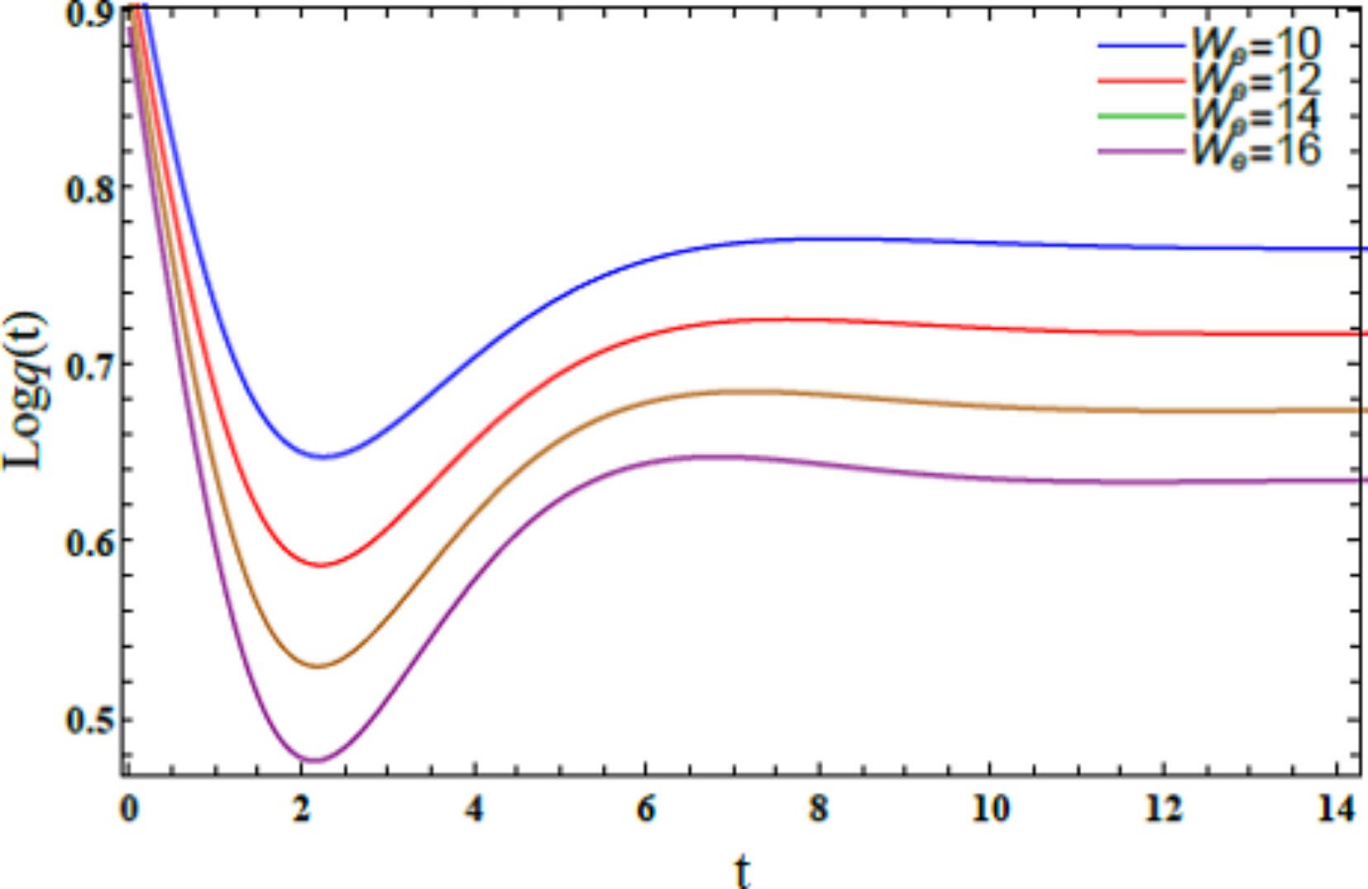
Elucidates $W_{e}$ on the stability area.

Fig. 15 demonstrates the graph of the solution $$Log\,q(t)$$ vs. time $$t$$ for various magnitudes of the Weber numeral $$W_{e} = 10,\,\,\,12,\,\,\,14,\,\,\,16$$. The other numerals are presumed as $$B = .5,\,\,\rho = 1,\,\,\mu = 1.5,$$$$B_{d} = 10{,}$$$$H_{0} = {2,}\,\,\,k = 0.3,$$$$D_{a} = 0.9,\,\,\,Ha^{2} = 10,$$$$B_{g} = 0.{1,}\,\,{\text{U}} = {1}{\text{.5}}{.}\,\,\,\mu_{e} = .5 \, \,\,{\text{and}}\,\,Z = 15$$. The diagram elucidates that the curves exhibit decreasing performance as both the Weber numeral $$\left( {W_{e}>> 1} \right)$$ and time increase. Specifically, the curves decline in amplitude and stability with higher Weber numerals, indicating that the system’s behavior becomes less stable over time as the influence of inertial forces grows relative to ST forces. Physically, as the Weber numeral enlarges, the system’s oscillations (or disturbances) become larger in amplitude and less stable over time. This suggests that the liquid interface becomes more prone to disruptions when inertial forces dominate over ST. ST typically acts as a stabilizing force, holding the liquid interface together, while inertial forces drive the system to break apart or deform. Managing the Weber number is critical in applications that involve liquid systems. Higher Weber numeral points out that the liquid’s surface is more susceptible to disturbances, such as the formation of droplets or waves. When inertial forces escalate, they overpower the ST. This causes instability at the liquid interface. For example, this impact is evident in the breakup of a jet into droplets in liquid sprays or the formation of bubbles in emulsions. This outcome has diverse implications in several areas. In industrial and agricultural spray systems, controlling droplet size and distribution is crucial. Higher Weber numerals in spray nozzles can cause uneven droplet formation. This leads to poor coverage in applications like painting or pesticide delivery. Engineers use stability analysis to design nozzles that balance inertial and surface tension forces to optimize droplet formation and system performance. The stability of thin liquid films is significant in processes such as coating surfaces (paints, varnishes, or protective layers). High Weber numerals can cause film rupture or irregularities, affecting the quality of the coating. By controlling the Weber numeral, manufacturers can ensure smooth and uniform film application. In food processing, cosmetics, or pharmaceuticals, stable emulsions (mixtures of two immiscible liquids) are necessary for product consistency. A higher Weber numeral could lead to emulsion instability, causing phase separation or droplet coalescence, which is undesirable in products like creams, lotions, or sauces^28,31^.

**Fig. 16:**
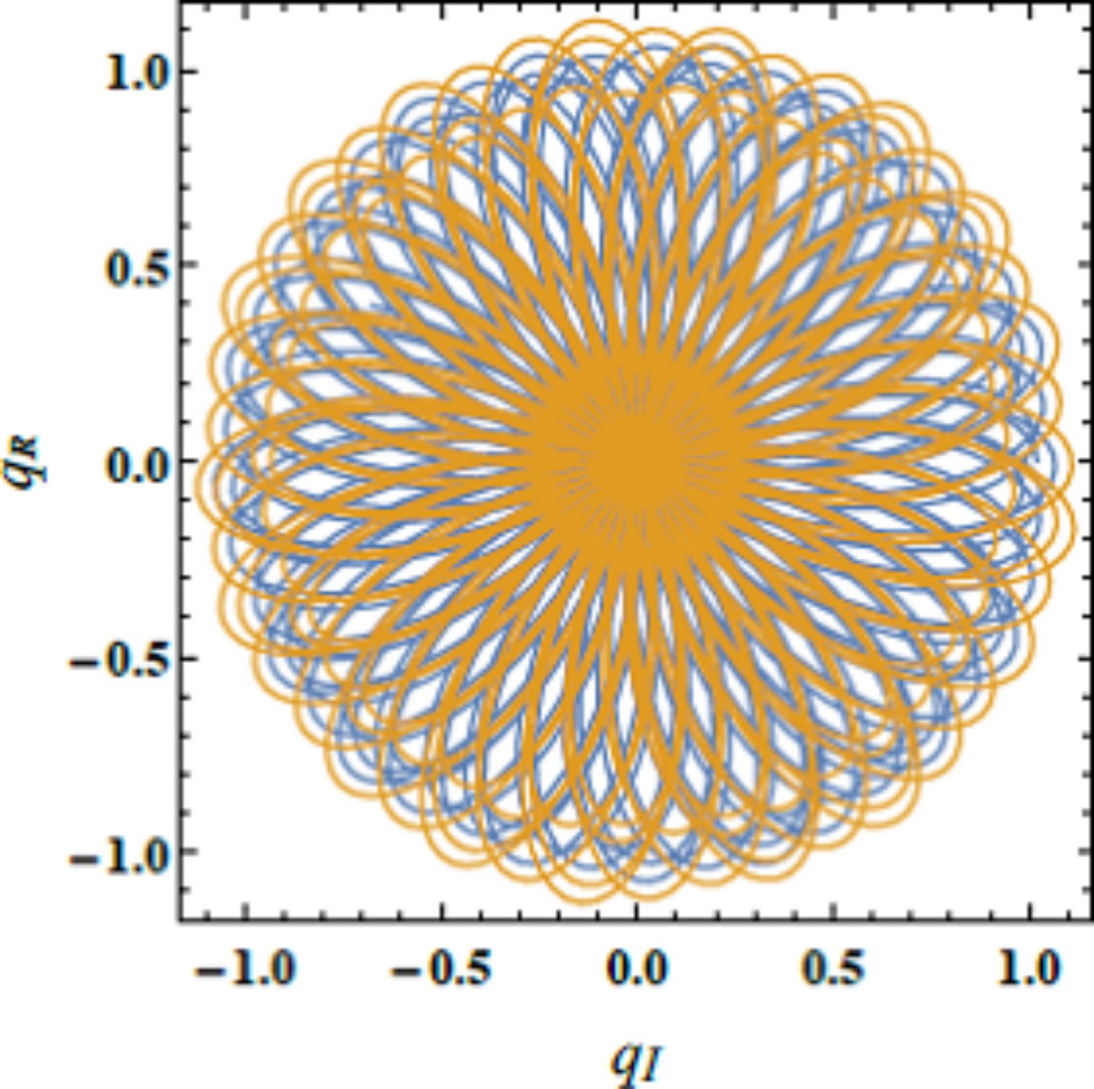
Expounds the Polar plot of $q_{R}$ vs. $q_{I}$.

Fig. 16 expounds the polar diagram of $q_{R}$ vs. $q_{I}$, which serves as a confirmation of the stability of the solution. The diagram visually reinforces that the system maintains its stability over time. As indicated by the consistent pattern observed in the PolarPlot. Physically, this stability suggests that the system’s dynamic behavior remains predictable and controlled under the given restrictions. This type of analysis is particularly relevant in fields. In robotics, stable control systems are crucial for ensuring precise movements and maintaining balance during tasks such as walking or manipulating objects. Instability in the system could result in erratic or unsafe movements. This makes stability analysis a key aspect of robotic design. In electronics, stable oscillations are vital for components like oscillators and timing circuits. These systems are essential for generating accurate signals and ensuring synchronization in devices such as clocks, communication systems, and computers. Instability here can cause system failures or incorrect data transmission. In biomedical engineering, stability analysis is critical for devices like pacemakers and prosthetics. Pacemakers must produce consistent electrical impulses to regulate heartbeats effectively, while prosthetic limbs rely on stable control systems to mimic natural movement, ensuring smooth and reliable operation for the user^28,31,32^.

**Fig. 17:**
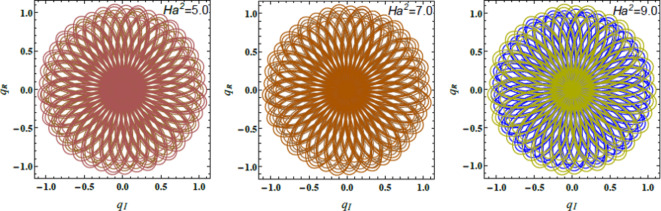
Describes $q_{R}$ vs. $q_{I}$ for $Ha^{2}$.

**Fig. 18:**
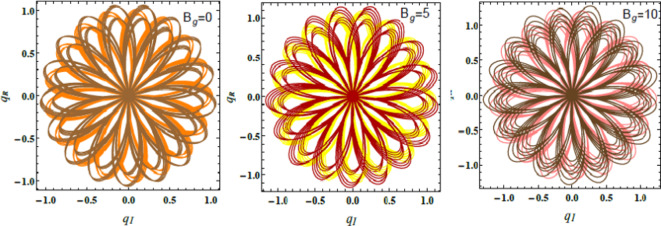
Explains $q_{R}$ vs. $q_{I}$ for $B_{g}$.

**Fig. 19:**
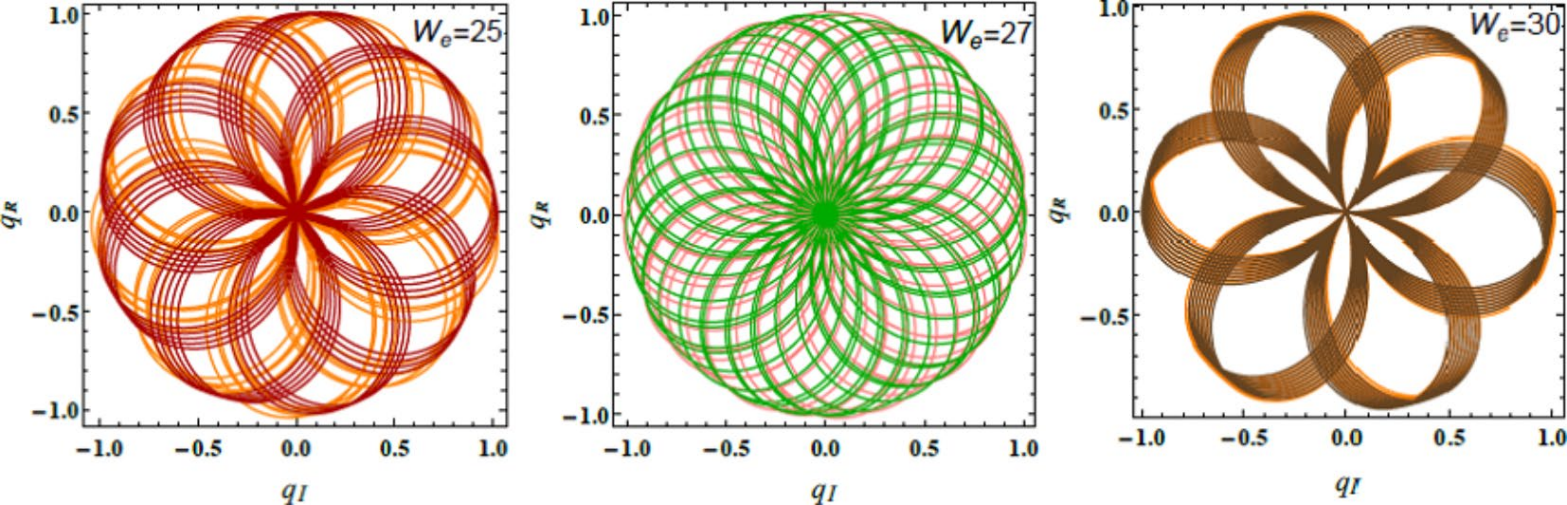
Displays $q_{R}$ vs. $q_{I}$ for $W_{e}$.

Figs. 17, 18 and 19 exhibit the NS for $$q_{R}$$ against $$q_{I}$$ employing the PolarPlot command, while varying the Hartmann numeral $$Ha^{2} ,$$ Bingham factor $$B_{g}$$, and Weber numeral $$W_{e}$$. The other numerals are presumed as $$\,B = 0.1,\,\,\rho = 0.2,\,\,\mu = .05,$$$${\text{U}} = {1}{\text{.5}}{.}$$$$H_{0} = {2,}\,$$$$B_{d} = {5}{\text{.0,}}$$$$D_{a} = .05,$$$$k = 0.5,$$$$\mu_{e} = 0.5 \, \,\,{\text{and}}\,\,\,\,Z = .01$$. These figures display the curves of symmetric patterns centered on specific points. The positions of these centers shift in response to changes in the Hartmann numeral, Bingham factor, and Weber numeral. These diagrams expound how key parameters directly influence the stability and behavior of liquid systems. The impacts of the Hartmann numeral, Bingham factor, and Weber numeral in these graphs are regarded. As these amounts enhance ordwindle. The curves either contract or expand, signifying changes in the system’s stability and dynamics. Physiologically, the Hartmann numeral reflects the influence of MFs on the liquid It has a stabilizing impact on the system by dampening disturbances in MHD applications like electromagnetic pumps and cooling systems. The Bingham factor relates to the material’s yield stress. That plays a role in systems where liquid must overcome a threshold force to flow. it is relevant in industrial processes involving BFs, like drilling mud or toothpaste. The Weber numeral highlights the balance between inertial forces and ST, affecting droplet formation in sprays and emulsions. That makes it critical in applications such as inkjet printing, coatings, and atomization processes. This detailed analysis aids improve the predictability and control of liquid performance. For example, in engineering, material science, and liquid processing systems. Thus, optimizing the stability is vital for enhancing performance and reliability^28,31,32,34^.

**Fig. 20:**
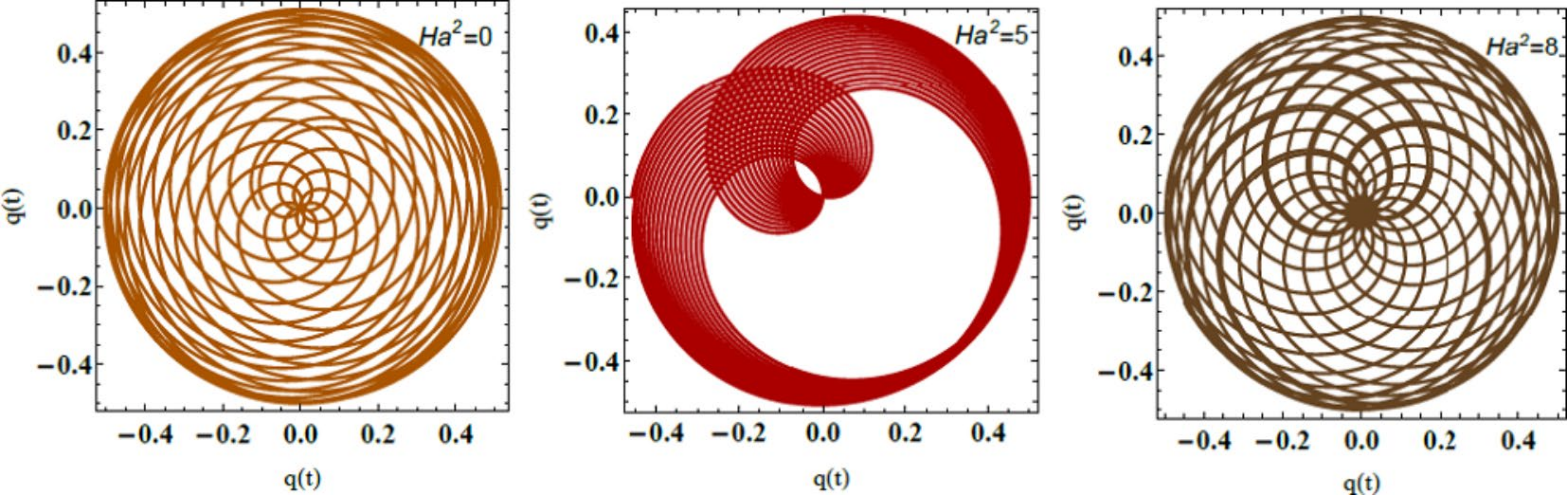
Elucidates $$q(t)$$ for $$Ha^{2}$$.

Finally, Fig. 20 demonstrates the PolarPlot of Eq. (51) for diverse amounts of the Hartmann numeral. This provides insight into the system’s performance under distinct MF intensities. As shown in the figure, the curves exhibit oscillations that either intensify or diminish as the Hartmann numeral $Ha^{2}$ elevates. These oscillations manifest as rotations along distinct closed or semi-closed elliptical paths, all converging around a central point. The performance highlighted here shows how magnetic forces, are quantified by the Hartmann numeral $Ha^{2}$. Physically magnetic forces play a crucial role in influencing the stability and inflow dynamics of liquid systems. As the Hartmann numeral elevates, the system becomes more stable. This indicates that MFs have a strong impact on resisting perturbations and disturbances in the flow. This stabilizing influence is essential in applications where precise magnetic control of liquid movement is required For example, in MHD generators, electromagnetic pumps, and medical devices that apply magnetic fluids, MFs dampen disturbances, enhancing stability. MFs enhance the stability and allow for fine-tuned control over the fluid’s conduct. This has significant implications in engineering and technology. Particularly in areas like plasma confinement in fusion reactors, where controlling liquid stability is critical. MFs can alter inflow patterns, enabling improved performance in processes like cooling systems. MF manipulation is necessary for efficient heat transfer. These findings emphasize the importance of magnetic control in liquid dynamics across diverse industrial and biomedical applications. Like, drug delivery systems applying ferrofluids or advanced manufacturing procedures involving liquid metal.^28,31,32^.

## Concluding remarks

In this work, the nonlinear stability of an interface concerning two BFs that fill with porous medium is deemed. A typical uniform and powerful perpendicular MF that considers the Lorentz force impacts the two liquids. With presenting ST at the interface, the two liquids exhibit distinct viscoelasticity, density, magnetic, and porosity medium properties. The contribution of viscoelasticity is only agreed at the surface of separation, also known as the VPT, because of the intricacy of the mathematical complexity. So, a collection of nonlinear BCs is estimated, while the flow-regulating equations are examined in a linear form. The nonlinear ODE of real and complex factors is altered into a linear one by applying the NPA relying on HFF. The nonlinear stability is discussed, and the interface presentation is closely scrutinized, utilizing the NPA. The NPA pursues to replace the nonlinear ODE with an equivalent linear one. The final formulation is a nonlinear PDE with complicated interface displacement factors. A comparison between the nonlinear ODE and its corresponding one in the light of the MS occurs to verify the novel methodology. Further dimensionless physical numerals are expressed via a non-dimensional analysis. A series of profiles are graphed to demonstrate and comprehend the characteristics of these statistics and how they affect the stability characteristics. The main key findings can be abridged as follows:

1. The existence of the Lorentz force in the momentum equation is regarded.
2. The VPT is utilized to effectively simplify the complexity of the problem, offering a clearer and more accessible analysis.
3. The NPA is applied to derive an alternative linear formulation, providing a novel perspective on the issue.
4. Even though the general case is quite complex, this article has effectively demonstrated and discussed its key aspects, providing clarity on the subject matter.
5. In this work, two cases were examined:
  i. Particular case (real coefficients) which occurs at Particular case (real coefficients) which occurs at $W_{e} = 0.$
    - In this case, it is found that $D_{a}$ has a destabilized influence. Meanwhile, both $Z$_&_ $Ha^{2}$ have a stabilized one.
  ii. General case (complex coefficients), which happens when $W_{e} \ne 0.$
    b. In this case, it is realized that the numerals $B_{g} ,\,\,Ha^{2}$ and $W_{e}$ have a stabilized influence on the stability configuration.
6. A group of PolarPlot diagrams for both cases ($W_{e} = 0.$ and $W_{e} \ne 0$) were exhibited.
7. Several Tables were meticulously designed to enhance the understanding of the stability mechanisms in the general case, specifically addressing the complex coefficients in the nonlinear characteristic equation governing interface displacement.
8. From a physical perspective, unstable solutions were considered undesirable; therefore, the PolarPlot corresponding to these solutions was deliberately omitted.

Overall, this study introduces innovative analytical techniques to address nonlinear instabilities in fluids, providing new insights into how MFs interact with viscoelastic behavior and Bingham plastic characteristics. The integration of advanced analytical methods improves the prediction and control of liquid instability, crucial for optimizing systems in various fields. The findings could lead to advancements in engineering, material science, and biological applications, such as medical devices and treatment methods for vascular diseases. As a progress work, the nonlinear stability analysis, in different geometries, in the case of the double interfaces will be studied.

## Electronic supplementary material

Below is the link to the electronic supplementary material.

Supplementary Material 1

## Data Availability

All data generated or analyzed during this study are included in this manuscript.

## List of symbols

$\,\underline{B}$: Magnetic induction vector
$B_{0j}$: Magnetic field strength
$$B_{d}$$: Bond numeral
$$B_{g}$$: Bingham parameter
$$C_{0j}$$: Integration constant
$$D_{a}$$: Darcy numeral
$\underline{E}$: Intensity of the electric field
$$\underline{g}$$: Acceleration gravity m s^-2^
$H_{0}^{2}$: Magnetic Bond numeral
$$Ha^{2}$$: Hartman numeral
$\underline{J}$: Electric current density
$$k$$: Wave numeral
$p_{j}$: Total perturbed pressure
$$t$$: Time
$$U_{j}$$: Uniform speed m s^-1^
$U$: The ratio of uniform velocities
$$\underline{V}$$: Velocity vector
$$W_{e}$$: Weber numeral
$$Z$$: Ohnesorge numeral

## Greek symbols

$\beta$: Permeability of media
$\dot{\gamma }$: Strain-rate tensor for the Bingham model
$$\mu_{i}$$: Kinematic viscosity
$\mu$: Ratio of kinematic viscosities
$$\mu_{ej}$$: Magnetic permeability
$\mu_{e}$: Ratio of magnetic permeabilities
$\rho_{j}$: Fluid density Kg m^-3^
$\rho$: Ratio of liquid densities
$$\sigma_{H}$$: Electrical conductivity S m^-1^
$\sigma_{ij}^{hydro}$: Hydrodynamic stress tensor
$$\tau_{0j}$$: Yield stress
$\tau_{ij}$: Cauchy stress tensor
$$\delta_{ij}$$: Kronecker delta
$$\Phi$$: Velocity potential
$$\Psi$$: Magnetic potential
