# Supplementary material for: Novel analytical perspectives on nonlinear instabilities of viscoelastic Bingham fluids in MHD flow fields
Source: Sci Rep. 2024 Nov 21;14:28843. doi: 10.1038/s41598-024-78848-8 (PMC11582699; doi:10.1038/s41598-024-78848-8)
Supplement: Supplementary file 1 — Supplementary Material 1 [file 41598_2024_78848_MOESM1_ESM.docx]

**Appendix**

The constants that appear in Eq. (34) may be listed as:

$r_{1}=b_{1}/b_{0}$, $r_{2}=b_{2}/b_{0}$, $r_{3}=b_{3}/b_{0}$, $r_{4}=b_{4}/b_{0}$, $r_{5}=b_{5}/b_{0}$, $r_{6}=b_{6}/b_{0}$, $r_{7}=b_{7}/b_{0}$, $r_{8}=b_{8}/b_{0}$, $r_{9}=b_{9}/b_{0}$, $r_{10}=b_{10}/b_{0}$, $r_{11}=b_{11}/b_{0}$, $r_{12}=b_{12}/b_{0}$, $r_{13}=b_{13}/b_{0}$ and $r_{14}=b_{14}/b_{0}$,

where

$b_{0}=\frac{1}{k}(\rho+1)$,$b_{1}=2 k Z (1+\mu)+\left(Z/k D_{a}\right)(1+\mu)+2Z Ha^{2}/\left(k\right),$ $b_{2}=\sqrt{W_{e}}\left(1+U \rho\right)$,

$b_{3}=B_{d}\left(1-\rho\right)-k^{2},-2k^{3}-k\mu_{e} \left(\mu_{e}-1\right)^{2}\left(1+\mu_{e}\right)^{-1}H_{0}^{2},$ $b_{4}=2 Z k^{2} \sqrt{W_{e}}\left(1+U \mu\right)+\frac{Z }{ D_{a}}\sqrt{W_{e}}\left(1+U \mu\right)+Z Ha^{2}\sqrt{W_{e}}\left(1+U \right)-2 Z B_{g} \sqrt{W_{e}} \left(1-\tau_{0}\right)$, $b_{5}=-(1-\rho)$, $b_{6}=2Z k^{2}\left(1-\mu\right)-\frac{Z }{ D_{a}}\left(1-\mu\right)$, $b_{7}=-k \sqrt{W_{e}}(1-\rho U)$,

$b_{8}=k^{2}Z B_{g} \sqrt{W_{e}} \left(1-\tau_{0}\right)-0.5 k^{2}\mu_{e}\left(1+\mu_{e}\right)^{-2}\left[\left(1-\mu_{e}\right)\left(3+2\mu_{e}+3\mu_{e}^{2}\right)+4\left(\mu_{e}-1\right)\right]H_{0}^{2},$ $b_{9}=2k^{3} Z \sqrt{ W_{e}} (1-\mu U)-k Z Ha^{2}\sqrt{W_{e}}(1-U)-k \frac{Z }{ D_{a}} \sqrt{W_{e}} (1-\mu U)$,$b_{10}=k\left(1+\rho\right)$, $b_{11}=4 k^{3} Z (1+\mu)+k \frac{Z }{ D_{a}} (1+\mu)+2k Z Ha^{2}$, $b_{12}= k^{2}\sqrt{W_{e}}\left(1+U \rho\right)$, $b_{13}=-\frac{3k^{4}}{2 }-\frac{3}{2}k^{3} \mu_{e}\left(\mu_{e}-1\right)^{2}\left(1+\mu_{e}\right)^{-1}H_{0}^{2}$ and$b_{14}=4 Z k^{4} \sqrt{W_{e}}\left(1+U \mu\right)+ k^{2} Z Ha^{2}\sqrt{W_{e}}(1+U)+\frac{Z }{ D_{a}} k^{2} \sqrt{W_{e}}\left(1+U \mu\right)-4 Z B_{g} k^{3}\sqrt{W_{e}} \left(1-\tau_{0}\right).$.

As well, $L_{1}\to L_{6}$ in Eqs. (64)-(66) are expressed as:

$L_{1}=r_{1}+\frac{B^{2}}{4}r_{11}$, $L_{2}=r_{2}+\frac{B^{2}}{4}r_{12}$, $L_{3}=r_{3}+\frac{3}{4}B^{2}(-\Delta^{2}r_{10}+r_{13})$, $L_{4}=r_{4}+\frac{3B^{2}}{4}r_{14}$,

$L_{5}=\frac{B^{2}}{4}(\Delta^{2} r_{5}+\Delta r_{6}+r_{8})$, and $L_{6}=\frac{B^{2}}{4}(\Delta r_{7}+r_{9})$.
